# Heated tobacco products for smoking cessation and reducing smoking prevalence

**DOI:** 10.1002/14651858.CD013790.pub2

**Published:** 2022-01-06

**Authors:** Harry Tattan-Birch, Jamie Hartmann-Boyce, Loren Kock, Erikas Simonavicius, Leonie Brose, Sarah Jackson, Lion Shahab, Jamie Brown

**Affiliations:** Department of Behavioural Science and HealthUniversity College LondonLondonUK; Nuffield Department of Primary Care Health SciencesUniversity of OxfordOxfordUK; Department of Addictions, Institute of Psychiatry, Psychology & NeuroscienceKing's College LondonLondonUK

## Abstract

**Background:**

Heated tobacco products (HTPs) are designed to heat tobacco to a high enough temperature to release aerosol, without burning it or producing smoke. They differ from e‐cigarettes because they heat tobacco leaf/sheet rather than a liquid. Companies who make HTPs claim they produce fewer harmful chemicals than conventional cigarettes. Some people report stopping smoking cigarettes entirely by switching to using HTPs, so clinicians need to know whether they are effective for this purpose and relatively safe. Also, to regulate HTPs appropriately, policymakers should understand their impact on health and on cigarette smoking prevalence.

**Objectives:**

To evaluate the effectiveness and safety of HTPs for smoking cessation and the impact of HTPs on smoking prevalence.

**Search methods:**

We searched the Cochrane Tobacco Addiction Group's Specialised Register, CENTRAL, MEDLINE, and six other databases for relevant records to January 2021, together with reference‐checking and contact with study authors and relevant groups.

**Selection criteria:**

We included randomised controlled trials (RCTs) in which people who smoked cigarettes were randomised to switch to exclusive HTP use or a control condition. Eligible outcomes were smoking cessation, adverse events, and selected biomarkers.  RCTs conducted in clinic or in an ambulatory setting were deemed eligible when assessing safety, including those randomising participants to exclusively use HTPs, smoke cigarettes, or attempt abstinence from all tobacco. Time‐series studies were also eligible for inclusion if they examined the population‐level impact of heated tobacco on smoking prevalence or cigarette sales as an indirect measure.

**Data collection and analysis:**

We followed standard Cochrane methods for screening and data extraction. Our primary outcome measures were abstinence from smoking at the longest follow‐up point available, adverse events, serious adverse events, and changes in smoking prevalence or cigarette sales. Other outcomes included biomarkers of harm and exposure to toxicants/carcinogens (e.g. NNAL and carboxyhaemoglobin (COHb)). We used a random‐effects Mantel‐Haenszel model to calculate risk ratios (RR) with 95% confidence intervals (CIs) for dichotomous outcomes. For continuous outcomes, we calculated mean differences on the log‐transformed scale (LMD) with 95% CIs. We pooled data across studies using meta‐analysis where possible.

**Main results:**

We included 13 completed studies, of which 11 were RCTs assessing safety (2666 participants) and two were time‐series studies. We judged eight RCTs to be at unclear risk of bias and three at high risk. All RCTs were funded by tobacco companies. Median length of follow‐up was 13 weeks.

No studies reported smoking cessation outcomes.

There was insufficient evidence for a difference in risk of adverse events between smokers randomised to switch to heated tobacco or continue smoking cigarettes, limited by imprecision and risk of bias (RR 1.03, 95% CI 0.92 to 1.15; I^2^ = 0%; 6 studies, 1713 participants). There was insufficient evidence to determine whether risk of serious adverse events differed between groups due to very serious imprecision and risk of bias (RR 0.79, 95% CI 0.33 to 1.94; I^2^ = 0%; 4 studies, 1472 participants). There was moderate‐certainty evidence for lower NNAL and COHb at follow‐up in heated tobacco than cigarette smoking groups, limited by risk of bias (NNAL: LMD −0.81, 95% CI −1.07 to −0.55; I^2^ = 92%; 10 studies, 1959 participants; COHb: LMD −0.74, 95% CI −0.92 to −0.52; I^2^ = 96%; 9 studies, 1807 participants). Evidence for additional biomarkers of exposure are reported in the main body of the review.

There was insufficient evidence for a difference in risk of adverse events in smokers randomised to switch to heated tobacco or attempt abstinence from all tobacco, limited by risk of bias and imprecision (RR 1.12, 95% CI 0.86 to 1.46; I^2^ = 0%; 2 studies, 237 participants). Five studies reported that no serious adverse events occurred in either group (533 participants). There was moderate‐certainty evidence, limited by risk of bias, that urine concentrations of NNAL at follow‐up were higher in the heated tobacco use compared with abstinence group (LMD 0.50, 95% CI 0.34 to 0.66; I^2^ = 0%; 5 studies, 382 participants). In addition, there was very low‐certainty evidence, limited by risk of bias, inconsistency, and imprecision, for higher COHb in the heated tobacco use compared with abstinence group for intention‐to‐treat analyses (LMD 0.69, 95% CI 0.07 to 1.31; 3 studies, 212 participants), but lower COHb in per‐protocol analyses (LMD −0.32, 95% CI −1.04 to 0.39; 2 studies, 170 participants). Evidence concerning additional biomarkers is reported in the main body of the review.

Data from two time‐series studies showed that the rate of decline in cigarette sales accelerated following the introduction of heated tobacco to market in Japan. This evidence was of very low‐certainty as there was risk of bias, including possible confounding, and cigarette sales are an indirect measure of smoking prevalence.

**Authors' conclusions:**

No studies reported on cigarette smoking cessation, so the effectiveness of heated tobacco for this purpose remains uncertain. There was insufficient evidence for differences in risk of adverse or serious adverse events between people randomised to switch to heated tobacco, smoke cigarettes, or attempt tobacco abstinence in the short‐term. There was moderate‐certainty evidence that heated tobacco users have lower exposure to toxicants/carcinogens than cigarette smokers and very low‐ to moderate‐certainty evidence of higher exposure than those attempting abstinence from  all tobacco. Independently funded research on the effectiveness and safety of HTPs is needed.

The rate of decline in cigarette sales accelerated after the introduction of heated tobacco to market in Japan but, as data were observational, it is possible other factors caused these changes. Moreover, falls in cigarette sales may not translate to declining smoking prevalence, and changes in Japan may not generalise elsewhere. To clarify the impact of rising heated tobacco use on smoking prevalence, there is a need for time‐series studies that examine this association.

## Plain language summary

**Do heated tobacco products help people to quit smoking, are they safe for this purpose, and have they led to falls in smoking rates?**

**Key messages**

Heated tobacco probably exposes people to fewer toxins than cigarettes, but possibly more than not using any tobacco. Falls in cigarette sales appeared to speed up following the launch of heated tobacco in Japan, but we are uncertain whether this is caused by people switching from cigarettes to heated tobacco.

We need more independently funded research into whether heated tobacco helps people stop smoking, whether it results in unwanted effects, and the impact of rising heated tobacco use on smoking rates.

**What are heated tobacco products? **

Heated tobacco products are designed to heat tobacco to a high enough temperature to release vapour, without burning it or producing smoke. They differ from e‐cigarettes because they heat tobacco leaf/sheet rather than a liquid. Many of the harmful chemicals in cigarette smoke are created by burning tobacco. So heating not burning tobacco could reduce the amount of chemicals a user ingests. Some people report stopping smoking cigarettes entirely by switching to using heated tobacco.

**Why we did this Cochrane Review **

Because cigarette smoking is addictive, many people find it difficult to stop despite the harm it causes. We aimed to find out whether trying to switch to heated tobacco helps people stop smoking cigarettes, and whether it results in unwanted effects. We also wanted to find out whether rising heated tobacco use has affected smoking rates or cigarette sales.

**What did we do?**

We looked for studies that reported on the use of heated tobacco for stopping smoking, and on unwanted effects and toxin exposure in people asked to use heated tobacco. Here we only included randomised controlled trials, where treatments were given to people at random. This type of study is considered the most reliable way of determining if a treatment works. Finally, we searched for studies looking at changes in smoking rates and cigarette sales following the launch of heated tobacco to market. We included studies published up to January 2021.

**What we found**

Our search found 13 relevant studies. No studies reported whether heated tobacco helps people stop smoking cigarettes. Eleven trials, all funded by tobacco companies and with 2666 adult smokers, compared unwanted effects and toxin levels in people randomly assigned to use heated tobacco or to continue smoking cigarettes or abstain from tobacco use.

Two studies looked at how trends in cigarette sales changed following the launch of heated tobacco in Japan.

**What are the results of our review? **

We do not know whether using heated tobacco helps people to stop smoking cigarettes (no studies measured this).

We are uncertain whether the chances of getting unwanted symptoms from being asked to use heated tobacco are different compared with cigarettes (6 studies, 1713 participants) or no tobacco (2 studies, 237 participants). Serious unwanted symptoms in the short time period studied (average 13 weeks) were rare in all groups, which means we are uncertain about any differences. Toxin levels were probably lower in people using heated tobacco than those smoking cigarettes (10 studies, 1959 participants), but may be higher than in people not using any tobacco products (5 studies, 382 participants).

The launch of heated tobacco products in Japan may have caused the decline in cigarette sales to speed up over time (two studies), but it is unclear whether the fall in the percentage of people who smoke also sped up because no studies looked at this.

**How reliable are these results?**

Results are based on data from a small number of studies, most of which were funded by tobacco companies.

Results on unwanted effects are likely to change as more evidence becomes available. However, we are moderately confident that levels of measured toxins are lower in people using heated tobacco than smoking cigarettes, but less confident that levels were higher than in people not using any tobacco. We are also less confident that the launch of heated tobacco caused the fall in cigarette sales to speed up, as results came from a single country.

## Summary of findings

**Summary of findings 1:** Heated tobacco use compared with cigarette smoking

**Heated tobacco use compared with cigarette smoking**						
**Patient or population:** people who smoke **Setting:** USA, Japan, UK, South Korea, Poland **Intervention:** heated tobacco use **Comparison:** cigarette smoking						
**Outcomes**	Anticipated absolute effects^*^ (95% CI)		**Relative effect (95% CI)**	**№ of participants (studies)**	**Certainty of the evidence (GRADE)**	**Comments**
	**Risk with cigarette smoking**	**Risk with heated tobacco use**				
**Adverse events** – measured by self‐report	**Study population**		**RR 1.03** (0.92 to 1.15)	1713 (6 RCTs)	⊕⊕⊝⊝ **Low**^a,b^	—
	235 per 1000	242 per 1000 (216 to 270)				
**Serious adverse events** – measured by self‐report and medical records	**Study population**		**RR 0.79** (0.33 to 1.94)	2009 (9 RCTs)	⊕⊝⊝⊝ **Very low**^a,c^	—
	13 per 1000	10 per 1000 (4 to 24)				
**NNAL** at follow‐up – measured in urine	—	—	**LMD 0.81** lower (1.07 lower to 0.55 lower)	1959 (10 RCTs)	⊕⊕⊕⊝ **Moderate**^a^	LMD has no units as it is calculated from the logarithm of biomarker measurements.
**COHb** at follow‐up – measured in blood	—	—	**LMD 0.74** lower (0.92 lower to 0.52 lower)	1807 (9 RCTs)	⊕⊕⊕⊝ **Moderate**^a^	LMD has no units as it is calculated from the logarithm of biomarker measurements.
*The risk in the intervention group (and its 95% confidence interval) is based on the assumed risk in the comparison group and the **relative effect** of the intervention (and its 95% CI). **CI:** confidence interval; **COHb:** carboxyhaemoglobin; **LMD:** difference in means of log‐transformed measurements; **NNAL:** 4‐(methylnitrosamino)‐1‐(3‐pyridyl)‐1‐butanol; **RCT:** randomised controlled trial; **RR:** risk ratio.						
**GRADE Working Group grades of evidence** **High certainty:** we are very confident that the true effect lies close to that of the estimate of the effect. **Moderate certainty:** we are moderately confident in the effect estimate: the true effect is likely to be close to the estimate of the effect, but there is a possibility that it is substantially different. **Low certainty:** our confidence in the effect estimate is limited: the true effect may be substantially different from the estimate of the effect. **Very low certainty:** we have very little confidence in the effect estimate: the true effect is likely to be substantially different from the estimate of effect.						

^a^Downgraded one level for risk of bias: all studies were at either unclear or high risk of bias. ^b^Downgraded one level for imprecision: confidence intervals contain clinically meaningful benefit and clinically meaningful harm. ^c^Downgraded two levels for imprecision: confidence intervals contain large clinically meaningful benefit and clinically meaningful harm.

**Summary of findings 2:** Heated tobacco use compared with abstinence from tobacco

**Heated tobacco use compared with abstinence from tobacco**						
**Patient or population:** people who smoke **Setting:** USA, Japan, UK, South Korea **Intervention:** heated tobacco use **Comparison:** abstinence from tobacco						
**Outcomes**	Anticipated absolute effects^*^ (95% CI)		**Relative effect (95% CI)**	**№ of participants (studies)**	**Certainty of the evidence (GRADE)**	**Comments**
	**Risk with abstinence from tobacco**	**Risk with heated tobacco use**				
**Smoking cessation** – not measured	—	—	—	—	—	—
**Adverse events** – measured by self‐report	**Study population**		**RR 1.12** (0.86 to 1.46)	237 (2 RCTs)	⊕⊝⊝⊝ **Very low**^a,b^	—
	468 per 1000	525 per 1000 (403 to 684)				
**Serious adverse events** – measured by self‐report and medical records	**Study population**		—	533 (5 RCTs)	⊕⊝⊝⊝ **Very low**^c,d^	No serious adverse events were reported.
	See comment	See comment				
**NNAL** at follow‐up – measured in urine	—	—	**LMD 0.50** higher (0.34 higher to 0.66 higher)	382 (5 RCTs)	⊕⊕⊕⊝ **Moderate**^d^	LMD has no units as it is calculated from the logarithm of biomarker measurements.
**COHb** at follow‐up – measured in blood	**LMD 0.69 higher** (0.07 higher to 1.31 higher) for analyses using intention‐to‐treat, but LMD 0.32 lower (1.04 lower to 0.39 higher) for per‐protocol analyses.			212 (3 RCTs)	⊕⊝⊝⊝ **Very low**^a,d,e^	Reported narratively due to inconsistency of results across subgroups.
*The risk in the intervention group (and its 95% confidence interval) is based on the assumed risk in the comparison group and the **relative effect** of the intervention (and its 95% CI). **CI:** confidence interval; **COHb:** carboxyhaemoglobin; **LMD:** difference in means of log‐transformed measurements; **NNAL:** 4‐(methylnitrosamino)‐1‐(3‐pyridyl)‐1‐butanol; **RCT:** randomised controlled trial; **RR:** risk ratio.						
**GRADE Working Group grades of evidence** **High certainty:** we are very confident that the true effect lies close to that of the estimate of the effect. **Moderate certainty:** we are moderately confident in the effect estimate: the true effect is likely to be close to the estimate of the effect, but there is a possibility that it is substantially different. **Low certainty:** our confidence in the effect estimate is limited: the true effect may be substantially different from the estimate of the effect. **Very low certainty:** we have very little confidence in the effect estimate: the true effect is likely to be substantially different from the estimate of effect.						

^a^Downgraded one level for imprecision: confidence intervals contained clinically meaningful benefit and clinically meaningful harm. ^b^Downgraded two levels for risk of bias: all studies were considered at high risk of bias. ^c^Downgraded two levels for imprecision: no serious adverse events occurred so confidence intervals could not be calculated. ^d^Downgraded one level for risk of bias: two of the five studies were considered high risk of bias, while three had uncertain risk of bias. ^e^Downgraded two levels for inconsistency: there was unexplained heterogeneity and results were inconsistent across subgroups and sensitivity analyses.

**Summary of findings 3:** Heated tobacco use compared with snus use

**Heated tobacco use compared with snus use**						
**Patient or population:** people who smoke **Setting:** USA **Intervention:** heated tobacco use **Comparison:** snus use						
**Outcomes**	Anticipated absolute effects^*^ (95% CI)		**Relative effect (95% CI)**	**№ of participants (studies)**	**Certainty of the evidence (GRADE)**	**Comments**
	**Risk with snus use**	**Risk with heated tobacco use**				
**Smoking cessation** – not measured	—	—	—	—	—	—
**Adverse events** – measured by self‐report	**Study population**		**RR 1.30** (0.94 to 1.80)	87 (1 RCT)	⊕⊝⊝⊝ **Very low**^a,b^	—
	558 per 1000	726 per 1000 (525 to 1000)				
**Serious adverse events** – measured by self‐report and medical records	**Study population**		Not estimable	44 (1 RCT)	⊕⊝⊝⊝ **Very low**^a,c^	No serious adverse events were reported.
	See comment	See comment				
**NNAL** at follow‐up – measured in urine	—	—	**MD 160 ng/24 hours lower** (339 lower to 19 higher)	50 (1 RCT)	⊕⊝⊝⊝ **Very low**^a,b^	—
**COHb** at follow‐up – measured in blood	6.0% saturation	3.75% saturation (2.5% higher to 5.0% higher)	**MD 2.24% saturation higher** (0.69 higher to 3.79 higher)	52 (1 RCT)	⊕⊕⊝⊝ **Low**^a^	—
*The risk in the intervention group (and its 95% confidence interval) is based on the assumed risk in the comparison group and the **relative effect** of the intervention (and its 95% CI). **CI:** confidence interval; **COHb:** carboxyhaemoglobin; **MD:** mean difference; **NNAL:** 4‐(methylnitrosamino)‐1‐(3‐pyridyl)‐1‐butanol; **RCT:** randomised controlled trial; **RR:** risk ratio.						
**GRADE Working Group grades of evidence** **High certainty:** we are very confident that the true effect lies close to that of the estimate of the effect. **Moderate certainty:** we are moderately confident in the effect estimate: the true effect is likely to be close to the estimate of the effect, but there is a possibility that it is substantially different. **Low certainty:** our confidence in the effect estimate is limited: the true effect may be substantially different from the estimate of the effect. **Very low certainty:** we have very little confidence in the effect estimate: the true effect is likely to be substantially different from the estimate of effect.						

^a^Downgraded two levels for indirectness: participants in the included study were given carbon‐tip heated tobacco products, which are unlike heated tobacco products currently on the market. ^b^Downgraded one levels for imprecision: confidence intervals incorporate no clinically meaningful difference. ^c^Downgraded two levels for imprecision: no serious adverse events occurred so confidence intervals could not be calculated.

**Summary of findings 4:** Population‐level impact of heated tobacco on cigarette smoking prevalence

**Population‐level impact of heated tobacco on cigarette smoking prevalence**			
**Patient or population:** N/A **Setting:** Japan **Intervention:** introduction of heated tobacco to market **Comparison:** N/A			
**Outcomes**	**Impact**	**№ of participants (studies)**	**Certainty of the evidence (GRADE)**
**Cigarette sales** – assessed with national and regional sales data	1 study found that the yearly percentage decline in cigarette sales accelerated after the introduction of heated tobacco in Japan, increasing from a mean decline of −3.10% across 2011–2015 to −16.38% across 2016–2019. A second study found similar results using a different method; it found that per capita cigarette sales were increasing at a rate of 0.10 to 0.14 (depending on statistical approach) per month before the introduction of heated tobacco in Japan. After the introduction, they declined at a rate of 0.63 to 0.66 cigarettes per month.	N/A (2 observational studies)	⊕⊝⊝⊝ **Very low**^a,b^
N/A: not applicable/available.			
**GRADE Working Group grades of evidence** **High certainty:** we are very confident that the true effect lies close to that of the estimate of the effect. **Moderate certainty:** we are moderately confident in the effect estimate: the true effect is likely to be close to the estimate of the effect, but there is a possibility that it is substantially different. **Low certainty:** our confidence in the effect estimate is limited: the true effect may be substantially different from the estimate of the effect. **Very low certainty:** we have very little confidence in the effect estimate: the true effect is likely to be substantially different from the estimate of effect.			

^a^Downgraded one level for indirectness: cigarette sales do not necessarily translate to reductions in smoking prevalence, as smokers may reduce the amount they smoke rather than stop smoking entirely. ^b^Downgraded one level for risk of bias: one study was considered to be at serious risk of bias, while the other was deemed at moderate risk.

## Background

### Description of the condition

Tobacco use kills eight million people each year, making it one of the leading preventable causes of death worldwide (GBD 2021). Approximately 90% of these deaths result from the most harmful form of tobacco consumption: smoking (Drope 2018). Therefore, reducing smoking prevalence is one of the most effective ways of improving population health (Holford 2014).

Although most smokers want to quit, smoking is highly addictive. Most people who make a serious attempt to quit fail, with only 3% to 10% still abstinent after one year (Hughes 2004; Jackson 2019a). Available treatments such as behavioural support, varenicline, and nicotine replacement therapy (NRT) improve the chance that these attempts will succeed (Cahill 2016; Hartmann‐Boyce 2018; Hartmann‐Boyce 2019; Hartmann‐Boyce 2021a). However, even with these treatments, success rates are typically under 25%, and many who try to quit do not use any support (Borland 2012; Jackson 2019b). There remains an urgent need to identify new, effective, and safer alternatives to cigarettes to reduce smoking prevalence.

### Description of the intervention

Heated (or heat‐not‐burn) tobacco products (HTPs) are designed to heat tobacco leaf/sheet to a high enough temperature to release nicotine‐infused aerosol, without burning it or producing smoke. Many of the toxic and carcinogenic products of cigarette smoking are formed during combustion. HTPs are marketed as less harmful and as alternatives to conventional cigarettes because they are engineered to avoid combustion (Mathers 2017).  The extent to which they help people quit smoking is largely unknown, and their impact on youth uptake to smoking is contentious (Czoli 2020). Therefore, it is unclear what impact HTPs will have on smoking prevalence across the population.

'Premier' was the first HTP made available for consumers. It resembled a cigarette, but the tobacco was not directly burned, instead it was heated by lighting a carbon‐tip (i.e. not electronic). Premier was introduced to test markets throughout the US by RJ Reynolds in 1988, but it was not widely used and was discontinued in 1989 (Stapleton 1998). In the early 2000s, RJ Reyolds introduced another carbon‐tip HTP, 'Eclipse', and they funded research to support marketing claims that it reduced health risks relative to cigarettes. A court case in the US succeeded in challenging these reduced risk claims, but trial evidence did suggest users of Eclipse had lower exposure to toxicants than people smoking cigarettes (Anderson 2008; Rennard 2002). The first electronic HTPs were produced by Philip Morris International (PMI). They introduced 'Accord' into the US in 1997 and a similar product, 'Heatbar', in Germany in 2007 (Elias 2018). While these products have both since been discontinued, they acted as predecessors to 'IQOS'. 

The current HTP market is dominated by electronic rather than carbon‐tip devices. Current brands include  IQOS by PMI, 'glo' by British American Tobacco, and 'Ploom Tech' by Japan Tobacco International (WHO 2018). IQOS and glo produce aerosol by directly heating tobacco sticks which resemble small cigarettes. Conversely, Ploom Tech produces aerosol by heating a similar liquid to that found in e‐cigarettes. This aerosol is then drawn through a bulb of tobacco to infuse it with flavour. Of these products, IQOS was the first to launch in 2014 in Japan and Italy, and it has since entered markets across Asia, Europe, and the Americas. Most recently, in 2019, the US Food and Drug Administration (FDA) permitted the sale of IQOS (FDA 2019) and in 2020 authorised their marketing as a modified‐exposure tobacco product (FDA 2020). At the time of writing, HTPs were most popular in Japan and the Republic of Korea; tobacco sticks for HTPs constituted 15.8% and 8.0% respectively of each country's tobacco market in 2018 (WHO 2018). Market research by Euromonitor estimates that HTPs had an increased share of the retail value of all nicotine or tobacco products between 2017 and 2018, which was similar to e‐cigarettes globally (Euromonitor 2020). However, HTP use remains rare in North America and much of Europe (Gallus 2021; Laverty 2021; Miller 2020; Tattan‐Birch 2021). 

### How the intervention might work

Nicotine is the primary addictive compound in cigarettes. Neuroadaptation to repeated nicotine delivery from smoking causes people who quit to experience withdrawal and cravings (Benowitz 2010; West 2017). Like cigarettes, HTPs contain nicotine. They may aid smoking cessation in a similar way to NRT and e‐cigarettes: people can use them to relieve nicotine cravings without smoking cigarettes (Wadgave 2016). HTPs may also provide certain advantages over NRT. One limitation of NRT is that it poorly addresses the behavioural and sensory cues associated with cigarette smoking, such as repeated hand‐to‐mouth actions and the scratch at the back of the throat when inhaling smoke. Evidence shows that denicotinised cigarettes reduce cravings and withdrawal symptoms among abstinent smokers, despite containing negligible levels of nicotine (Rose 2006). This suggests that these cues contribute to cigarette dependence. HTPs may more closely replicate these cues than NRT. Because HTP aerosol is delivered to the throat and lungs, nicotine absorption likely occurs more rapidly than from patches, gum, or lozenges, which are absorbed through the skin or buccal mucosa (Simonavicius 2018). The speed with which nicotine is absorbed may be one of the key determinants of dependence (Benowitz 2009), so HTPs may provide a better replacement for cigarette smoking than NRT. E‐cigarettes also deliver nicotine rapidly to the throat and possibly lungs (Hajek 2020; Wagener 2017) and, like HTPs, they mimic the hand‐to‐mouth actions of cigarette smoking. But only HTPs contain tobacco leaf/sheet, so their flavour may more closely resemble cigarette smoke (Poynton 2017), which may make them more attractive to smokers (Tompkins 2021). Moreover, in some countries, the sale of nicotine e‐cigarettes is banned or heavily restricted (Dyer 2019). In such environments, HTPs may be the only consumer product available that delivers nicotine rapidly through a potentially less harmful medium than tobacco smoke*. *

We refer to the complete replacement of cigarettes with HTPs as 'switching'. A substantial proportion of people who use HTPs for smoking cessation may continue using these products for some time after they stop smoking cigarettes, as is the case with e‐cigarettes (Hajek 2019; Simonavicius 2020). Encouraging people to switch from smoking cigarettes to using HTPs would only be beneficial if HTPs are less harmful to health or if HTPs eventually help people taper off nicotine entirely. The safety of HTPs to users depends on both the acute harm, measured by adverse and serious adverse events, and the long‐term harm of repeated inhalation of damaging compounds in HTP aerosols.

Biomarkers can be used to measure exposure to these harmful toxicants and carcinogens. Important exposure biomarkers include: 4‐(methylnitrosamino)‐1‐(3‐pyridyl)‐1‐butanol (NNAL), a marker of tobacco‐specific N‐nitrosamine exposure that is linked to numerous cancers (IARC 2012); 1‐hydroxypyrene (1‐OHP) and 1‐ and 2‐naphthol, indicators of exposure to polycyclic aromatic hydrocarbons that are associated with cancers and kidney and liver damage; 3‐hydroxypropylmercapturic acid (3‐HPMA), a marker of exposure to acrolein that is linked to respiratory disease (Yeager 2016); and carboxyhaemoglobin (COHb), a measure of recent carbon monoxide (CO) intake. Details about these and other exposure biomarkers are available in Appendix 1.

Manufacturers of HTPs claim that the aerosol they produce contains significantly lower levels of toxicants than cigarette smoke and, as a result, that they have reduced risk potential or are less harmful (BAT 2020; PMI 2018). Two systematic reviews supported claims about lower toxicant levels, but found that most research into HTPs was funded through sources affiliated with the tobacco industry (Jankowski 2019; Simonavicius 2018). In addition, reduced exposure does not necessarily indicate reduced harm. The US FDA judged that there was sufficient evidence that IQOS reduced exposure to harmful chemicals (FDA 2020), but insufficient evidence on whether switching from smoking to HTPs reduces harm, such as pulmonary function or biomarkers linked to smoking‐related harm (Glantz 2018; Moazed 2018). It is also the case that safety, especially of longer‐term use, cannot be addressed with confidence until long‐term cohort studies have collected sufficient data.

### Why it is important to do this review

There is substantial variation between countries in their regulatory approaches to HTPs, and within countries across different nicotine products. In order for policymakers to regulate HTPs effectively and proportionately, there is a need for evidence to inform a judgement on their likely public health impact. The net impact of HTPs on public health will depend on a variety of factors. Three influential elements that could result in HTPs benefiting public health are if they increase smoking cessation, decrease smoking prevalence, and are less harmful than cigarette smoking. Conversely, even if these products are shown to be much less harmful than cigarettes, HTPs could damage public health if they hinder smoking cessation or increase smoking prevalence.

The effect of HTP use on smoking prevalence will depend on whether they influence rates of attempted quitting among cigarette smokers, the proportion of these attempts that are successful, cigarette uptake among non‐smokers, and relapse among people who had previously quit smoking. Therefore, we are not only interested in studies that report individual‐level effects of HTPs on smoking cessation, but also those that estimate their population‐level effects on smoking prevalence*.* This review will investigate up‐to‐date evidence for both, using appropriate study designs.

The growing popularity of HTPs means that people who smoke may be increasingly likely to seek advice from practitioners who need to know whether HTPs are effective for smoking cessation and how their safety compares with cigarettes and other alternative nicotine products. If HTPs are found to be safe and effective for smoking cessation, they would offer a novel treatment for cigarette addiction. Moreover, evidence on associations between HTP use and smoking prevalence will help to guide the regulation of HTPs.

Licensed smoking cessation medications tend to be used for a short time while quitting, whereas people may continue using HTPs for extended periods after they quit. This means that it is especially important to evaluate indicators of the long‐term safety of HTP use (such as exposure to toxicants and carcinogens) in addition to adverse events occurring in the short term.

## Objectives

To evaluate the effectiveness and safety of HTPs for smoking cessation and the impact of HTPs on smoking prevalence.

## Methods

### Criteria for considering studies for this review

#### Types of studies

We divided the methods into the three subsections, representing the different objectives of the review: effectiveness for smoking cessation, safety, and smoking prevalence.

##### Effectiveness for smoking cessation

Individual‐level and cluster‐randomised controlled trials (RCTs) to examine the effectiveness (or efficacy) of HTPs for tobacco smoking cessation.

##### Safety

Individual‐level, randomised cross‐over and cluster‐RCTs to explore adverse and serious adverse events and biomarkers of toxicant and carcinogen exposure. RCTs in optimised settings for smoking cessation, such as those where participants stayed in a clinic with restricted access to tobacco products, were eligible for inclusion, as were studies in naturalistic or ambulatory settings. 

##### Smoking prevalence

Interrupted and multiple time‐series studies were included to examine the population‐level effect of HTPs on cigarette smoking prevalence. Smoking cessation interventions may not be representative of the way most people use HTPs, which is without support from a researcher or trained specialist. Moreover, even if HTPs encourage smoking cessation among those trying to quit, their impact on smoking prevalence depends on how they affect smoking initiation and the number of people who make a quit attempt and are successful in remaining abstinent. We used time‐series studies to assess how changes in HTP prevalence are associated with changes in smoking prevalence (or cigarette sales), with the limitation that associations might not reflect causal effects.

We included studies regardless of language or status of publication.

#### Types of participants

##### Effectiveness and safety

We included adults who were defined as current cigarette smokers by the study at the time of enrolment.

##### Smoking prevalence

We did not restrict by participant characteristics, as we are interested in population‐level data. We focused on any individuals who indicated their smoking status or consumption and HTP use or consumption, measured by survey or by record of sales.

#### Types of interventions

HTPs, defined as hand‐held devices that aim to heat tobacco to a temperature high enough to produce a nicotine‐infused aerosol but too low to cause self‐sustaining combustion. HTPs differ from e‐cigarettes in that they heat compressed tobacco leaf rather than a liquid that is infused with nicotine.

##### Effectiveness and safety

We were interested in studies that compared HTPs, or the addition of HTPs, to no treatment (i.e. continued tobacco smoking), placebo or any other smoking cessation treatment, including NRT, e‐cigarettes, snus, varenicline, bupropion, and behavioural support. HTPs could be provided in addition to any other smoking cessation treatment, providing there was equivalent provision of the additional treatment for the control group. We only included studies where participants in the HTP arm were instructed to stop smoking combustible cigarettes for at least seven days. 

##### Smoking prevalence

For interrupted time‐series studies, the interventions of interest were the introduction of HTPs to market or the time point where HTPs began gaining popularity. For multiple time‐series studies, we were interested in the extent to which changes in the prevalence of HTP use were associated with changes in the prevalence of cigarette smoking (or cigarette sales as a proxy), after adjusting for other influences that could affect changes in the prevalence of smoking at the population level. 

#### Types of outcome measures

##### Primary outcomes

###### Effectiveness

- Tobacco smoking cessation at the longest follow‐up point available, using intention‐to‐treat and biochemically verified abstinence where possible. While HTPs contain tobacco, they are designed to avoid or minimise combustion and smoke. Therefore, HTP use was not classified as tobacco smoking. If review updates find studies reporting smoking cessation, we will only include those which report abstinence at four‐week follow‐up or longer. We will use the strictest definition of abstinence recorded, that is, prolonged or continuous abstinence over point prevalence, and biochemically verified over self‐reported abstinence. Typically, Cochrane Tobacco Addiction Group reviews only include data on smoking cessation at six months or longer. We will include short‐term outcomes in the next update of this review because we anticipate a paucity of longer‐term data. In subsequent updates, as and when more data become available, we may change the inclusion criteria accordingly.

###### Safety

- Number of people reporting adverse events and serious adverse events. We defined serious adverse events as medical incidents that are potentially life‐threatening, require hospitalisation, result in disability or death, or a combination of these. Adverse events were medical problems — including cough, headache, and dry mouth — that did not fulfil the above criteria to be considered serious.

###### Smoking prevalence

- Change in the prevalence of cigarette smoking, measured as the proportion of people in a given locality that regularly smoke cigarettes or other combustible tobacco products, over a defined time period. We included cigarette sales as a proxy for prevalence, measured as the number of cigarettes sold in a given locality over a given time period. This was used as a proxy because, in a population where mean cigarette consumption among smokers remains stable, declines in cigarette sales imply falls in smoking prevalence. However, it should be considered an indirect measure of prevalence because smokers can reduce their cigarette consumption without quitting.

##### Secondary outcomes

All secondary outcomes are measures of **safety**. We only included studies that reported safety outcomes at one‐week follow‐up or longer. 

- Biomarkers of toxicant and carcinogen exposure. We included measures of exposure to tobacco‐specific N‐nitrosamines, polycyclic aromatic hydrocarbons, volatile organic compounds, and CO (see Appendix 1 for details on associations with health outcomes).
- Biomarkers of harm, also known as surrogate endpoints. We included measures of lung function (forced expiratory volume in one second (FEV_1_), forced vital capacity (FVC), and FEV_1_/FVC), blood pressure, heart rate, heart rate variability, and blood oxygen saturation.

### Search methods for identification of studies

#### Electronic searches

We searched the following databases on 19 January 2021:

- Cochrane Tobacco Addiction Group's Specialised Register (for details of how this register is populated see the Cochrane Tobacco Addiction Group's website: tobacco.cochrane.org/resources/cochrane-tag-specialised-register);
- Cochrane Central Register of Controlled Trials (CENTRAL; 2020, Issue 12);
- MEDLINE (OvidSP);
- Embase (OvidSP);
- PsycINFO (OvidSP);
- Business Source Complete;
- Factiva;
- ClinicalTrials.gov;
- World Health Organization International Clinical Trials Registry Platform (ICTRP) (apps.who.int/trialsearch/).

We restricted the search to studies published since 2008, three years before the first internet searches for HTPs began (Google Trends 2020).

The search terms were:

heated tobacco OR carbon‐heated tobacco OR heat‐not‐burn OR heat not burn OR tobacco heating system$ OR tobacco heating device$ OR tobacco heating product$ OR tobacco vapor product$ OR tobacco vapour product$. We also searched for the term smoking AND (iqos OR glo OR ploom OR ifuse OR fuse OR pulze OR teeps OR pax OR mok OR lil OR iuoc OR htp OR thp OR ths OR chtp).

As we were only interested in studies that used humans, we excluded those with the terms animal$ OR mice OR rat$ OR in vitro OR in silico OR in vivo in their title.

#### Searching other resources

We searched the reference lists of eligible studies found in the literature search.

In order to identify government reports and in‐press or unpublished studies, we contacted relevant charities and authors of published research or trial protocols. We used the searches of ClinicalTrials.gov and the ICTRP detailed above to identify trial registry records.

### Data collection and analysis

#### Selection of studies

Two review authors (of HTB, JB, LK, ES, and LB) independently prescreened titles and abstracts of articles identified in the search, using a screening checklist. We resolved disagreements through discussion or referral to a third review author. We conducted screening using Covidence software (Covidence).

Two review authors (of HTB, JB, LK, ES, and LB) independently screened the full text of articles that passed prescreening. We consulted a third review author to resolve any disagreements that were not resolved through discussion.

#### Data extraction and management

We produced two custom data extraction forms: one for effectiveness and safety, and the other for smoking prevalence. Details of these forms are available in Appendix 2.

Two review authors (of HTB, JB, LK, ES, and LB) independently extracted data from included studies. When discrepancies could not be resolved through discussion, we referred to a third review author. We contacted authors of included studies if additional information was needed.

#### Assessment of risk of bias in included studies

##### Effectiveness and safety

Two review authors (of HTB, JB, LK, ES, and LB) independently assessed risks of bias for all included RCTs using the Cochrane risk of bias tool version 1. We followed the guidance as set out in the *Cochrane Handbook for Systematic Reviews of Interventions* to evaluate the following domains: sequence generation; allocation concealment; blinding of outcome assessment; incomplete outcome data; selective reporting; and other sources of bias (Higgins 2011). 

##### Smoking prevalence

Two review authors independently assessed risk of bias for included time‐series studies using the ROBINS‐I tool (Sterne 2016).

#### Measures of treatment effect

##### Effectiveness and safety

We calculated risk ratios (RRs) and 95% confidence intervals (CIs) for dichotomous outcomes.

For continuous safety data, we calculated mean differences on the raw (MD) or log‐transformed (LMD) scale and the corresponding 95% CIs between the heated tobacco and control groups at follow‐up. When studies reported geometric means, we converted these onto the (natural) log scale, and when studies being pooled reported mixtures of geometric and arithmetic means, we converted them all onto the log scale, using Method 1 described in Higgins 2008 where appropriate.

We used the longest follow‐up data reported, with treatment effects calculated on an intention‐to‐treat basis where possible.

##### Smoking prevalence

For interrupted time‐series studies, the treatment effect could have been reflected by the step change and change in trends in smoking prevalence or cigarette sales following the introduction of HTPs to the market (or the time point where they started gaining popularity), after adjusting for confounding variables.

For multiple time‐series studies (in future review updates), the treatment effect of interest will be the association between HTP prevalence and smoking prevalence or cigarette sales, after adjusting for confounding variables. Where variables are log‐transformed, the resulting coefficient describes the percentage change in cigarette smoking prevalence associated with a 1% change in HTP prevalence.

#### Unit of analysis issues

##### Effectiveness and safety

For RCTs with more than two intervention arms, we combined data from all relevant intervention conditions where HTPs were offered. For RCTs with more than two control arms, we combined data from each of these arms, and we chose the most appropriate comparator. If it is not appropriate to pool the intervention arms (in future updates) then we will split the control arm to act as a comparator to each separate intervention arm. If future updates of this review identify cluster‐RCTs, we will attempt to extract an estimate of the effect that accounts for the cluster design of the study. Where this is not reported, we will attempt to perform the correct analysis if required data are available.

#### Dealing with missing data

##### Effectiveness

If we assess smoking cessation in future updates of this review, we will assume that people with missing data at follow‐up have not stopped smoking, as is common in the field.

##### Safety

When assessing adverse and serious adverse events, we calculated the proportion of those available at follow‐up who experienced an event (when such data are available) rather than the proportion of people who were randomised, when follow‐up information was reported. When assessing biomarkers, we removed participants with missing follow‐up data from the analysis.

##### Smoking prevalence

We did not expect issues with missing data in time‐series studies.

#### Assessment of heterogeneity

To assess whether to conduct meta‐analyses, we considered the characteristics of included studies to identify substantial clinical or methodological heterogeneity. If we deemed the studies to be sufficiently homogeneous to be combined meaningfully, we assessed statistical heterogeneity using the I^2^ statistic. If the I^2^ statistic was greater than 50%, we reported substantial heterogeneity. If I^2^ was greater than 75%, we considered the appropriateness of presenting pooled results, and based this decision on consistency in the direction of effect across included studies.

#### Assessment of reporting biases

In future updates of this review, we will assess reporting bias using funnel plots if we deem it appropriate to pool 10 or more studies in any analysis. The greater the asymmetry in the plots, the higher the risk of reporting bias.

#### Data synthesis

##### Effectiveness

The primary outcome of smoking cessation provides dichotomous data. Following the standard methods of the Cochrane Tobacco Addiction Group, we aimed to combine RRs and 95% CIs from individual studies using a Mantel‐Haenszel random‐effects model, to calculate pooled overall RRs with 95% CIs.

##### Safety

For dichotomous safety outcomes (i.e. adverse and serious adverse events), we combined RRs and 95% CIs from individual studies using a Mantel‐Haenszel random‐effects model to calculate pooled overall RRs with 95% CIs.

For continuous safety outcomes measuring biomarkers, we pooled the MDs or LMDs and measures of variance of individual studies using a generic inverse variance random‐effects model.

##### Smoking prevalence

We aimed to calculate pooled estimates and their standard errors using a random‐effects model for each of three coefficients, when reported: step change in smoking prevalence or cigarette sales following the introduction of HTPs; change in these trends after the introduction; and changes associated with changes in prevalence or sale of HTPs. We did not pool time‐series studies with notably different time periods (e.g. weekly versus annual).

#### Subgroup analysis and investigation of heterogeneity

For biomarker outcomes, we undertook subgroup analyses to investigate differences by whether analyses were per‐protocol or intention‐to‐treat. We define per‐protocol analyses as those that only included participants who exclusively (or almost exclusively) used the product they were assigned, whereas intention‐to‐treat analyses include all participants regardless of actual product use.

If appropriate for future updates of this review, we will undertake subgroup analyses to investigate differences by:

- intensity of behavioural support provided;
- characteristics of HTP device (e.g. model used).

#### Sensitivity analysis

We aimed to carry out sensitivity analyses removing studies:

- judged at high risk of bias for at least one domain;
- with a minimum length of follow‐up of less than four weeks (safety outcomes only);
- where participants were given carbon‐tip, rather than electronic, HTPs.

If appropriate for future updates of this review, we will also carry out sensitivity analyses where we:

- remove studies that are funded by (or authors have received funding from) the tobacco industry;
- only classify participants as HTP users if they use their product daily (smoking prevalence only);
- only include interrupted time‐series studies in localities where HTPs achieved widespread use after they were introduced to market.

#### Summary of findings and assessment of the certainty of the evidence

We created summary of findings tables using GRADEpro GDT for all primary outcomes and for two biomarkers of exposure (NNAL and COHb), following the guidelines in *Cochrane Handbook of Systematic Reviews of Interventions* (GRADEpro GDT; Higgins 2021; Schünemann 2020). We chose NNAL and COHb because they are well‐established indicators of tobacco smoke exposure (Chang 2017; Hedblad 2005). We used the five GRADE considerations (risk of bias, inconsistency, imprecision, indirectness, and publication bias) to assess the certainty of the body of evidence for each of these outcomes. 

## Results

### Description of studies

#### Results of the search

Our bibliographic database searches identified 1504 non‐duplicate records (Figure 1). We found a further four records through screening references in the papers identified through electronic searches. We screened all records and retrieved the full‐text of 121 potentially relevant articles. After screening and checking the full texts, we included 23 records, representing 13 completed (Characteristics of included studies) and three ongoing studies (Characteristics of ongoing studies). We excluded 98 records during full text screening, and we give reasons for exclusion for 11 studies (Characteristics of excluded studies).

**1:**
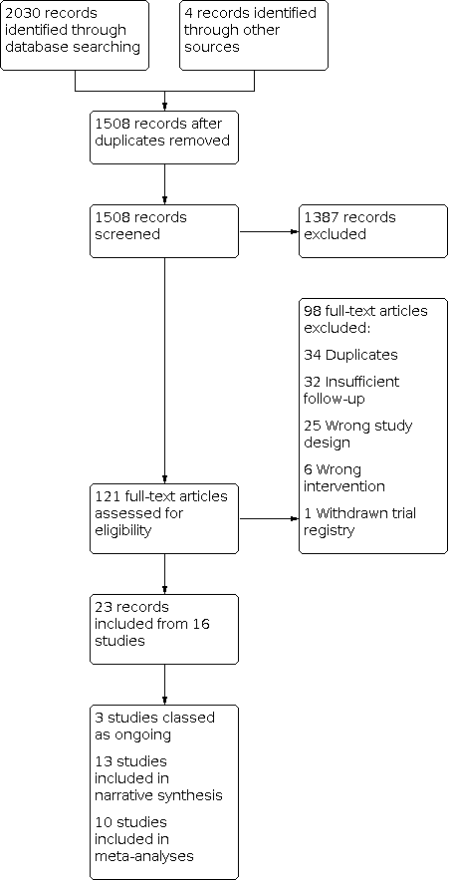


#### Included studies

A summary of the 13 included studies is given below. Further details of each study can be found in the Characteristics of included studies section.

##### Participants

Of the 13 included studies, 11 collected data from participants. Two studies used sales data and are thus excluded from subsequent discussion of participant characteristics. A total of 2666 participants were recruited across the 11 RCTs. Three studies were conducted in Japan, three in the USA, two in Poland, two in the UK, and one in South Korea. These studies were conducted in adults who smoked cigarettes. Seven studies exclusively recruited participants who were not motivated to quit smoking cigarettes. One study only recruited participants diagnosed with generalised chronic periodontitis (NCT03364751). Three studies only recruited people who were Japanese or of "Japanese ethnicity" (Lüdicke 2018; NCT03364751; Tricker 2012b), while Martin 2012 only recruited those of "Caucasian ethnicity". Participants stayed in confinement in a clinic for the duration of the trial in three studies (Tricker 2012a; Tricker 2012b; Tricker 2012c). Another three studies started with a confinement period of five days, before moving to an ambulatory setting for the rest of the trial (Bosilkovska 2020; Haziza 2019; Lüdicke 2018). The remaining five studies used an ambulatory setting with regular clinical visits. Median follow‐up length was 13 weeks, and three studies had less than four weeks of follow‐up (Tricker 2012a; Tricker 2012b; Tricker 2012c).

##### Interventions and comparators

All 11 included RCTs gave HTPs to participants. Two studies provided participants with the carbon‐tip products 'CHTP 1.2' and 'Eclipse' (Bosilkovska 2020; Ogden 2015). All others provided electronic heating devices alongside tobacco sticks, with PMI's IQOS‐family products (or their predecessors) provided in eight studies and BAT's glo‐family products in one study (Gale 2020).

All 11 RCTs compared participants randomised to receive a HTP or to continue smoking cigarettes. Five studies also had tobacco abstinence as an additional comparator and one study had snus use as an additional comparator (Ogden 2015). Summaries of study results by comparator are available in Effects of interventions. Further details on the intervention and comparator groups for each are available in the Characteristics of included studies section.

There were two interrupted time‐series studies using cigarette sales data from Japan. The intervention in these studies was the introduction of heated tobacco to market, with the launch of IQOS in 2015 or 2016 (depending on region). 

##### Outcomes

Of the 13 included studies:

- none reported smoking cessation rates;
- 10 reported data on adverse events (four of which did not provide data in each trial arm). Commonly reported adverse events included cough, headache, gastrointestinal issues (e.g. diarrhoea), dry mouth, hyperglycaemia, and decreased haemoglobin;
- 10 reported data on serious adverse events. Most studies defined serious adverse events as medical incidents that were potentially life‐threatening, require hospitalisation, resulted in disability or death, of a combination of these;
- 11 reported data on at least one biomarker of toxicant and carcinogen exposure;
- five reported data on at least one biomarker of harm;
- none reported time‐series data on smoking prevalence;
- two reported time‐series data on cigarette sales.

##### Study types and funding

Eleven studies were RCTs and two were observational time‐series studies. All 11 RCTs were funded by the tobacco industry. One time‐series study was funded through government grants, while the other had no specific funding.

#### Excluded studies

Figure 1 shows the most common reasons for exclusion of studies during full‐text screening, which were: duplicate reports; less than one week of follow‐up; and wrong study design (e.g. testing acute rather than extended effects of HTP use).

In the Characteristics of excluded studies table, we list more detailed exclusion reasons for 11 of these studies. This list is not comprehensive, only containing studies that a reader might plausibly expect be included. 

### Risk of bias in included studies

Overall, we judged eight of the 11 included RCTs at unclear risk of bias and three at high risk of bias, assessed using the ROB v1 criteria (Higgins 2011). Figure 2 shows judgements across the risk of bias domains for each RCT. Detailed rationale for these judgements can be found in the Characteristics of included studies.

**2:**
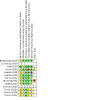
review authors' judgements about risk of bias domains for each included RCT study. Risk of bias for time‐series studies (Cummings 2020; Stoklosa 2020), assessed using ROBINS‐I tool, are shown in Appendix 3 and Appendix 4.

Risk of bias for the two included time‐series studies was assessed using the ROBINS‐I tool (Sterne 2016). One time‐series study was at moderate risk of bias, while the other was at serious risk. Detailed risk of bias assessments for these time‐series studies can be found in the appendices (Appendix 3; Appendix 4).

#### Allocation

All included RCTs were at unclear risk of selection bias, as there was no or insufficient information about random sequence generation or allocation concealment, or both. 

#### Blinding

We judged all studies at low risk of detection bias, as most reported outcomes were biochemical and hence judged at low risk of differential misreport. We planned to assess performance bias for smoking cessation outcomes, with studies judged at low risk if intervention and control arms received similar levels of behavioural support. As no study reported on smoking cessation outcomes, performance bias was not assessed.

#### Incomplete outcome data

Seven studies were at low risk of attrition bias, due to high and similar rates of follow‐up across treatment and comparator arms (Bosilkovska 2020; Gale 2020; Lüdicke 2018; Lüdicke 2019; Martin 2012; NCT03364751; Ogden 2015). Three studies were at unclear risk as they did not provide sufficient details about attrition (Tricker 2012a; Tricker 2012b; Tricker 2012c). Haziza 2019 was at high risk of attrition bias due to substantial loss to follow‐up that was greater in the heated tobacco arm.

#### Selective reporting

We judged five studies at low risk of reporting bias, as all prespecified outcomes were reported (Bosilkovska 2020; Gale 2020; Haziza 2019; Lüdicke 2019; NCT03364751). Five studies were at unclear risk as there was no preregistered study protocol (Martin 2012; Ogden 2015; Tricker 2012a; Tricker 2012b; Tricker 2012c). Lüdicke 2018 was at high risk of reporting bias, as one preregistered outcome of interest was not reported (FEV_1_/FVC).

#### Other potential sources of bias

One study was at high risk of other bias as it did not report results across randomised trial arms (NCT03364751). Instead, they only reported results based on actual product use.

### Effects of interventions

See: Table 1; Table 2; Table 3; Table 4

See: Table 1: heated tobacco use compared with cigarette smoking; Table 2: heated tobacco use compared with abstinence from tobacco; Table 3: heated tobacco use compared with snus use; Table 4: population‐level impact of heated tobacco on cigarette smoking prevalence.

Data on each outcome are summarised below, alongside links to forest plots. In these forest plots, benefit of HTPs is usually shown on the left, as lower toxicant levels or risk of adverse events indicates benefits of HTPs relative to the comparator. 

#### Effectiveness

##### Tobacco smoking cessation

No studies reported on the effectiveness of heated tobacco for smoking cessation.

#### Safety

##### Heated tobacco use versus cigarette smoking

###### Adverse events

Pooled data from six studies showed insufficient evidence of a difference in the number of participants reporting **adverse events **between those in the heated tobacco use and cigarette smoking groups, but the CI contained the possibility of small but clinically meaningful differences in both directions (RR 1.03, 95% CI 0.92 to 1.15; I^2^ = 0%; 1713 participants; Analysis 1.1; Table 1). Two studies were at high risk of bias, while the remaining four were at unclear risk. Removing studies judged at high risk of bias did not substantially change the interpretation of results (RR 0.98, 95% CI 0.87 to 1.11; I^2^ = 0%; 1472 participants), neither did removing the two studies that used carbon‐tip, rather than electronic, HTPs (RR 1.04, 95% CI 0.82 to 1.30; I^2^ = 35%; 1510 participants). All six studies had a follow‐up of at least four weeks.

###### Serious adverse events

Pooled data from four studies showed insufficient evidence of a difference in **serious adverse events** reported in the heated tobacco use compared with cigarette smoking group, with a wide CI that contained no difference as well as the possibility of more events in either group (RR 0.79, 95% CI 0.33 to 1.94; I^2^ = 0%; 1472 participants; Analysis 1.2; Table 1). All pooled studies were at unclear risk of bias and had a follow‐up of at least four weeks. Removing the two studies that used carbon‐tip, rather than electronic, HTPs did not substantially change the interpretation of results (RR 0.93, 95% CI 0.34 to 2.58; I^2^ = 0%; 1269 participants). In a further five studies, there were no serious adverse events reported, which meant their data could not be pooled (Haziza 2019; Lüdicke 2018; Tricker 2012a; Tricker 2012b; Tricker 2012c).

###### Secondary outcomes

####### Toxicant and carcinogen exposure

Pooled data from 1960 participants across 10 studies showed: 

- lower **1‐OHP** at follow‐up in heated tobacco use compared with cigarette smoking groups (LMD −0.42, 95% CI −0.67 to −0.17; Analysis 1.3). Heterogeneity was high at I^2^ = 94%, but the direction of the difference was consistent across all studies except Ogden 2015, where carbon‐tip HTPs were provided. It was also consistent across sensitivity analyses removing two studies at high risk of bias, two studies using carbon‐tip HTPs, and three studies with less than four weeks of follow‐up (Table 5);
- lower **3‐HPMA** at follow‐up in heated tobacco use compared with cigarette smoking groups (LMD −0.40, 95% CI −0.62 to −0.17; Analysis 1.8). Heterogeneity was high at I^2^ = 95%, but the direction of the difference was consistent across sensitivity analyses and all studies except Ogden 2015 (Table 5);
- lower **MHBMA** at follow‐up in heated tobacco use compared with cigarette smoking groups (LMD −1.15, 95% CI −1.52 to −0.78; Analysis 1.9). Heterogeneity was high at I^2^ = 94%, but the direction of the difference was consistent across studies and sensitivity analyses (Table 5);
- lower **NNAL** at follow‐up in heated tobacco use compared with cigarette smoking groups (LMD −0.81, 95% CI −1.07 to −0.55; Analysis 1.10; Table 1). Heterogeneity was high at I^2^ = 92%, but the direction of the difference was consistent across sensitivity analyses and all studies except Ogden 2015 (Table 5). Another study also reported NNAL; as data were analysed based on actual product use rather than randomised group, it was not pooled (NCT03364751). It found results that were compatible with those from pooled data (LMD −1.46, 95% CI −1.81 to −1.10; 151 participants).

**1:** Sensitivity analyses for heated tobacco use versus cigarette smoking

**Outcomes**	**All data**			**No high risk of bias**			**Only electronic devices**			**≥ 4 weeks' follow‐up**		
	**No. of participants (studies)**	**MD (95% CI)**	**I^2^ statistic**	**No. of participants (studies)**	**MD (95% CI)**	**I^2^ statistic**	**No. of participants (studies)**	**MD (95% CI)**	**I^2^ statistic**	**No. of participants (studies)**	**MD (95% CI)**	**I^2^ statistic**
**Biomarkers of exposure **												
1‐OHP^a^	1960 (10)	−0.42 (−0.67 to −0.17)	94%	1764 (8)	−0.40 (−0.70 to −0.10)	95%	1805 (8)	−0.54 (−0.75 to −0.34)	90%	1664 (7)	−0.28 (−0.57 to 0.00)	93%
1‐Naphthol	63 (1)	2.60μg/24 hours (−16.11 to 21.31)	N/A	63 (1)	2.60μg/24 hours (−16.11 to 21.31)	N/A	None	N/A	N/A	63 (1)	2.60μg/24 hours (−16.11 to 21.31)	N/A
2‐Naphthol	63 (1)	−4.00μg/24 (−7.89 to −0.11)	N/A	63 (1)	−4.00μg/24 (−7.89 to −0.11)	N/A	None	N/A	N/A	63 (1)	−4.00μg/24 (−7.89 to −0.11)	N/A
Exhaled CO	1322 (3)	−9.13ppm, (−10.49 to −7.78)	4%	1322 (3)	−9.13ppm, (−10.49 to −7.78)	4%	1322 (3)	−9.13ppm, (−10.49 to −7.78)	4%	1322 (3)	−9.13ppm, (−10.49 to −7.78)	4%
COHb^a^	1807 (9)	−0.74 (−0.97 to −0.52)	96%	1611 (7)	−0.76 (−1.07 to −0.44)	97%	1659 (7)	−0.84 (−1.07 to −0.60)	96%	1511 (6)	−0.24 (−0.36 to −0.12)	95%
3‐HPMA^a^	1960 (10)	−0.40 (−0.62 to −0.17)	95%	1764 (8)	−0.34 (−0.59 to −0.09)	95%	1805 (8)	−0.43 (−0.63 to −0.22)	93%	1664 (7)	−0.48 (−0.80 to −0.16)	96%
Lead	None	N/A	N/A	None	N/A	N/A	None	N/A	N/A	None	N/A	N/A
Cadmium	None	N/A	N/A	None	N/A	N/A	None	N/A	N/A	None	N/A	N/A
MHBMA^a^	1960 (10)	−1.15 (−1.52 to −0.78)	94%	1764 (8)	−1.05 (−1.46 to −0.65)	94%	1805 (8)	−1.17 (−1.57 to −0.77)	94%	1664 (7)	−1.26 (−1.77 to −0.75)	96%
NNAL^a^	1959 (10)	−0.81 (−1.07 to −0.55)	92%	1963 (8)	−0.70 (−0.96 to −0.44)	92%	1805 (8)	−0.85 (−1.08 to −0.62)	89%	1663 (7)	−0.80 (−1.16 to −0.44)	94%
**Biomarkers of harm**												
FEV_1_^a^	1290 (5)	0.02 (0.00 to 0.03)	0%	1095 (3)	0.02 (0.01 to 0.03)	0%	1201 (4)	0.02 (0.00 to 0.03)	0%	1290 (5)	0.02 (0.00 to 0.03)	0%
FVC	196 (2)	−0.12 (−0.45 to 0.21)	38%	None	N/A	N/A	196 (2)	−0.12 (−0.45 to 0.21)	38%	196 (2)	−0.12 (−0.45 to 0.21)	38%
FEV_1_/FVC	None	N/A	N/A	None	N/A	N/A	None	N/A	N/A	None	N/A	N/A
Systolic blood pressure^a^	288 (3)	0.00 (−0.02 to 0.02)	0%	92 (1)	0.01 (−0.02 to 0.05)	N/A	196 (2)	−0.01 (−0.04 to 0.02)	0%	288 (3)	0.00 (−0.02 to 0.02)	0%
Diastolic blood pressure^a^	288 (3)	−0.00 (−0.03 to 0.03)	0%	92 (1)	0.02 (−0.03 to 0.07)	N/A	196 (2)	−0.02 (−0.06 to 0.02)	0%	288 (3)	−0.00 (−0.03 to 0.03)	0%
Heart rate	None	N/A	N/A	None	N/A	N/A	None	N/A	N/A	None	N/A	N/A
Blood oxygen saturation	None	N/A	N/A	None	N/A	N/A	None	N/A	N/A	None	N/A	N/A

^a^Difference in means calculated on log‐scale. 1‐OHP: 1‐hydroxypyrene; 3‐HPMA: 3‐hydroxypropylmercapturic acid; CI: confidence interval; CO: carbon monoxide; COHb: carboxyhaemoglobin; FEV_1_: forced expiratory volume in one second; FVC: forced vital capacity; MD: mean difference; MHBMA: monohydroxy‐3‐butenyl mercapturic acid; N/A: not available; NNAL: 4‐(methylnitrosamino)‐1‐(3‐pyridyl)‐1‐butanol.

Pooled data for nine studies showed lower levels of **COHb** at follow‐up in heated tobacco use compared with cigarette smoking groups (LMD −0.74, 95% CI −0.97 to −0.52; 1807 participants; Analysis 1.7; Table 1). Heterogeneity was high at I^2^ = 96%, but estimates from each study were consistently in favour of the heated tobacco group. Results were similar after removing two studies at high risk of bias, two studies using carbon‐tip HTPs, and three studies with less than four weeks of follow‐up (Table 5).

In addition, pooled data from three studies showed lower levels of **exhaled CO** at follow‐up in heated tobacco use compared with cigarette smoking groups (MD −9.13ppm, 95% CI −10.49 to −7.78; 1322 participants; Analysis 1.6). There was low heterogeneity at I^2^ = 4% and effects for each study were in the same direction. All three studies were at unclear risk of bias, used electronic HTPs, and had at least four weeks of follow‐up.

Ogden 2015 reported data from 63 participants showing insufficient evidence of a difference in **1‐naphthol** between the heated tobacco use and cigarette smoking groups, with the CI containing the possibility of clinically meaningful effects in either direction (MD 2.60 μg/24 hours, 95% CI −16.11 to 21.31; Analysis 1.4). The study also found that **2‐naphthol** was lower in the heated tobacco use group compared with the cigarette smoking group; however, the CIs were wide (MD −4.00 μg/24 hours, 95% CI −7.89 to −0.11; Analysis 1.5). This study was at unclear risk of bias, used a carbon‐tip HTP, and had a follow‐up of greater than four weeks. 

No studies reported on exposure to **lead** or **cadmium**.

####### Biomarkers of harm

Pooled data from five studies showed greater lung function, measured using**FEV_1_**, at follow‐up among participants in the heated tobacco use compared with cigarette smoking groups (LMD 0.02, 95% CI 0 to 0.03; I^2^ = 0%; 1290 participants; Analysis 1.11). Results were similar after removing two studies at high risk of bias and one study using carbon‐tip HTPs. All five studies had a follow‐up of at least four weeks (Table 5).

Pooled data from 196 participants across two studies found no evidence of a difference in **FVC** between those randomised to heated tobacco use versus cigarette smoking, but the CI contained the possibility of clinically meaningful differences in both directions (MD −0.12 L, 95% CI −0.45 to 0.21; I^2^ = 38%; Analysis 1.14). Both studies had at least four weeks of follow‐up, were judged at high risk of bias, and provided electronic rather than carbon‐tip devices.

Pooled data from 288 participants across three studies showed no evidence of a difference in **systolic blood pressure **(LMD 0.00, 95% CI −0.02 to 0.02; I^2^ = 0%; Analysis 1.12) or **diastolic blood pressure** (LMD 0.00, 95% CI −0.03 to 0.03; I^2^ = 0%; Analysis 1.13) at follow‐up between heated tobacco use and cigarette smoking groups. Results were similar after removing two studies at high risk of bias and one study using carbon‐tip HTPs. All three studies had a follow‐up of at least four weeks (Table 5).

No studies reported on **FEV_1_/FVC**, **heart rate**, or **blood oxygen saturation**.

##### Heated tobacco use versus abstinence from tobacco 

###### Adverse events

Pooled data from two studies showed insufficient evidence of a difference in the number of participants reporting **adverse events** between the heated tobacco use and attempted tobacco abstinence groups, with the CI containing the possibility of clinically meaningful differences in both directions (RR 1.12, 95% CI 0.86 to 1.46; I^2^ = 0%; 237 participants; Analysis 2.1; Table 2). Both studies were at high risk of bias, used electronic HTPs, and had a follow‐up of at least four weeks.

###### Serious adverse events

Five studies reported that no **serious adverse events** occurred across either the heated tobacco or tobacco abstinence groups (Haziza 2019; Lüdicke 2018; Tricker 2012a; Tricker 2012b; Tricker 2012c), which meant that data could not be pooled (533 participants; Analysis 2.2, Table 2). Two studies were at high risk of bias, while the remaining three were at unclear risk. All studies used electronic HTPs and two had at least four weeks of follow‐up. 

###### Secondary outcomes

####### Toxicant and carcinogen exposure

All five studies reporting on biomarkersof toxicant and carcinogen exposure for this comparison used electronic rather than carbon‐tip HTPs. Pooled data from 382 participants across these studies showed: 

- higher **1‐OHP** at follow‐up in heated tobacco use groups compared with tobacco abstinence groups, but CIs were wide and contained no difference (LMD 0.12, 95% CI −0.03 to 0.28; Analysis 2.3). Heterogeneity was moderate with an I^2^ of 54%, which reduced to 12% in a sensitivity analysis where the two studies at high risk of bias were removed. The direction of the effect was unchanged after removing these studies and after removing three studies with less than four weeks of follow‐up (Table 6);
- inconsistent results for **COHb** across subgroups, with I^2^ = 77% for subgroup differences. Subgroup results showed higher COHb in heated tobacco use compared with tobacco abstinence groups for intention‐to‐treat analyses (LMD 0.69, 95% CI 0.07 to 1.31; I^2^ = 96%; 3 studies, 212 participants; Analysis 2.4), but lower COHb, limited by imprecision, for per‐protocol analyses (LMD −0.32, 95% CI −1.04 to 0.39; I^2^ = 91%; 2 studies, 170 participants; Analysis 2.4). Because of these subgroup differences and high overall heterogeneity (I^2^ = 99%), we did not present pooled results (Table 2). Heterogeneity was 96% when we removed the two studies at high risk of bias and 91% when we removed the three studies with less than four weeks of follow‐up. The direction of the difference was reversed when studies with less than four weeks of follow‐up were removed (Table 6);
- higher **3‐HPMA** in heated tobacco use compared with tobacco abstinence groups (LMD 0.56, 95% CI 0.33 to 0.80; Analysis 2.5). Heterogeneity was high with an I^2^ of 85%, which reduced to 0% when removing three studies with less than four weeks of follow‐up. Differences were smaller when we removed these studies (LMD 0.35, 95% CI 0.20 to 0.50; 170 participants), but larger when we removed two studies at high risk of bias (LMD 0.64, 95% CI 0.32 to 0.96; 212 participants) (Table 6);
- higher **MHBMA** in heated tobacco use compared with tobacco abstinence groups (LMD 0.67, 95% CI −0.12 to 1.45; Analysis 2.6), but CIs contained the potential for no difference. Heterogeneity was high with an I^2^ of 96%, which reduced to 0% when removing three studies with less than four weeks of follow‐up. Differences were smaller when we removed these studies (LMD 0.07, 95% CI −0.16 to 0.30; 170 participants), but larger when we removed two studies at high risk of bias (LMD 0.97, 95% CI 0.02 to 1.92; 212 participants);
- higher **NNAL** in heated tobacco use compared with tobacco abstinence groups (LMD 0.50, 95% CI 0.34 to 0.66; I^2^ = 0%; Analysis 2.7; Table 2). Results were similar in sensitivity analyses removing two studies at high risk of bias and three studies with less than four weeks of follow‐up.

**2:** Sensitivity analyses for heated tobacco use versus abstinence from tobacco

**Outcomes**	**All data**			**No high risk of bias**			**≥ 4 weeks' follow‐up**		
	**No. of participants (studies)**	**MD (95% CI)**	**I^2^ statistic**	**No. of participants (studies)**	**MD (95% CI)**	**I^2^ statistic**	**No. of participants (studies)**	**MD (95% CI)**	**I^2^ statistic**
**Biomarkers of exposure **									
1‐OHP^a^	382 (5)	0.12 (−0.03 to 0.28)	54%	212 (3)	0.11 (−0.03 to 0.25)	12%	170 (2)	0.22 (−0.32 to 0.75)	84%
1‐Naphthol	None	N/A	N/A	None	N/A	N/A	None	N/A	N/A
2‐Naphthol	None	N/A	N/A	None	N/A	N/A	None	N/A	N/A
Exhaled CO	None	N/A	N/A	None	N/A	N/A	None	N/A	N/A
COHb^a^	382 (5)	0.30 (−0.40 to 1.00)	99%	212 (3)	0.69 (0.07 to 1.31)	97%	170 (2)	−0.32 (−1.04 to 0.39)	91%
3‐HPMA^a^	382 (5)	0.56 (0.33 to 0.80)	85%	212 (3)	0.64 (0.32 to 0.96)	89%	170 (2)	0.35 (0.20 to 0.50)	0%
Lead	None	N/A	N/A	None	N/A	N/A	None	N/A	N/A
Cadmium	None	N/A	N/A	None	N/A	N/A	None	N/A	N/A
MHBMA^a^	382 (5)	0.67 (−0.12 to 1.45)	96%	212 (3)	0.97 (0.02 to 1.92)	96%	170 (2)	0.07 (−0.16 to 0.30)	0%
NNAL^a^	382 (5)	0.50 (0.34 to 0.66)	0%	212 (3)	0.42 (−0.01 to 0.85)	0%	170 (2)	0.51 (0.34 to 0.69)	0%
**Biomarkers of harm**									
FEV_1_^a^	170 (2)	−0.00 (−0.06 to 0.06)	38%	None	N/A	N/A	170 (2)	−0.00 (−0.06 to 0.06)	38%
FVC	172 (2)	−0.02 (−0.29 to 0.26)	0%	None	N/A	N/A	172 (2)	−0.02 (−0.29 to 0.26)	0%
FEV_1_/FVC	None	N/A	N/A	None	N/A	N/A	None	N/A	N/A
Systolic blood pressure^a^	170 (2)	0.02 (−0.01 to 0.05)	0%	None	N/A	N/A	170 (2)	0.02 (−0.01 to 0.05)	0%
Diastolic blood pressure^a^	170 (2)	0.00 (−0.04 to 0.04)	0%	None	N/A	N/A	170 (2)	0.00 (−0.04 to 0.04)	0%
Heart rate	None	N/A	N/A	None	N/A	N/A	None	N/A	N/A
Blood oxygen saturation	None	N/A	N/A	None	N/A	N/A	None	N/A	N/A

^a^Difference in means calculated on the log‐scale. 1‐OHP: 1‐hydroxypyrene; 3‐HPMA: 3‐hydroxypropylmercapturic acid; CI: confidence interval; CO: carbon monoxide; COHb: carboxyhaemoglobin; FEV_1_: forced expiratory volume in one second; FVC: forced vital capacity; MD: mean difference; MHBMA: monohydroxy‐3‐butenyl mercapturic acid; N/A: not available; NNAL: 4‐(methylnitrosamino)‐1‐(3‐pyridyl)‐1‐butanol.

No studies reported on exposure to **1‐naphthol**, **2‐naphthol**, **exhaled CO**, **lead**, or **cadmium**.

####### Biomarkers of harm

Both of the studies that reported on biomarkers of harm were at high risk of bias, used electronic rather than carbon‐tip HTPs, and had at least four weeks of follow‐up. Pooled data from 170 participants across these two studies showed: 

- insufficient evidence of a difference in lung function, measured using**FEV_1_** at follow‐up, among participants in the heated tobacco use compared with tobacco abstinence groups, with the CI including the possibility of clinically meaningful differences in both directions (LMD −0, 95% CI −0.06 to 0.06; I^2^ = 38%; Analysis 2.8);
- higher **systolic blood pressure** at follow‐up in the heated tobacco use compared with tobacco abstinence groups, but the CI included no difference (LMD 0.02, 95% CI −0.01 to 0.05; I^2^ = 0%; Analysis 2.9);
- insufficient evidence of a difference in **diastolic blood pressure** at follow‐up between heated tobacco use and tobacco abstinence groups, with the CIs including the possibility of clinically meaningful differences in both directions (LMD 0, 95% CI −0.04 to 0.04; I^2^ = 0%; Analysis 2.10).

Both studies also reported data from 172 participants on **FVC**, with insufficient evidence for a difference between those randomised to use heated tobacco versus tobacco abstinence (MD −0.02 L, 95% CI −0.29 to 0.26;  I^2^ = 0%; Analysis 2.11). The CIs contained the possibility of clinically meaningful differences in both directions. 

No studies reported **FEV_1_/FVC**, **heart rate**, or **blood oxygen saturation**.

##### Heated tobacco use versus snus use

###### Adverse events

In Ogden 2015, a higher number of participants reported **adverse events** in the group assigned to use heated tobacco compared with snus, but the CI was wide and included no difference (RR 1.30, 95% CI 0.94 to 1.80; 87 participants; Analysis 3.1; Table 3). The study had a follow‐up of at least four weeks, was at unclear risk of bias, and used carbon‐tip HTPs.

###### Serious adverse events

Ogden 2015 reported that no **serious adverse events** occurred across either the heated tobacco or snus use groups (87 participants; Analysis 3.2; Table 3).

###### Secondary outcomes

####### Toxicant and carcinogen exposure

Data from 50 participants (52 participants for COHb) in Ogden 2015 showed:

- higher **1‐OHP** at follow‐up in the heated tobacco compared with snus group (MD 252 μg/24 hours, 95% CI 100 to 404; Analysis 3.3);
- insufficient  evidence of a difference in **1‐naphthol** between the heated tobacco and snus groups, but the CI was wide and it contained the possibility of clinically meaningful effects in either direction (MD −2.4 μg/24 hours, 95% CI −27.7 to 22.9; Analysis 3.4);
- lower **2‐naphthol** at follow‐up in the heated tobacco compared with snus group, but the CI was wide and contained no difference as well as the possibility of clinically meaningful effects in either direction (MD −3.4 μg/24 hours, 95% CI −10.4 to 3.6; Analysis 3.5);
- higher **COHb** at follow‐up in the heated tobacco compared with snus group (MD 2.24% saturation, 95% CI 0.69 to 3.79; Analysis 3.6; Table 3);
- higher **3‐HPMA** at follow‐up in the heated tobacco compared with snus group (MD 1.07 mg/24 hours, 95% CI 0.39 to 1.75; Analysis 3.7);
- insufficient evidence of a difference in **MHBMA **between the heated tobacco and snus groups, with the CI containing the possibility of clinically meaningful effects in either direction (MD 0.33 μg/24 hours, 95% CI −1.36 to 2.02; Analysis 3.8);
- lower **NNAL** at follow‐up in the heated tobacco compared with snus group, but the CI was wide and contained no difference (MD −160 ng/24 hours, 95% CI −339 to 19; Analysis 3.9, Table 3).

No studies reported on exposure to **exhaled CO**, **lead**, or **cadmium**.

####### Biomarkers of harm 

No studies reported on **FEV_1_**, **FVC**,**FEV_1_/FVC**, **systolic blood pressure**, **diastolic blood pressure**, **heart rate**, or **blood oxygen saturation**.

#### Smoking prevalence

##### Cigarette sales

Cummings 2020 found that the yearly percentage decline in **cigarette sales** accelerated after the introduction of HTPs in Japan, increasing from a mean decline of −3.10% across 2011–2015 to −16.38% across 2016–2019 (Table 4). This study was considered at serious risk of bias due to the limited number of time points (five) used to calculate the pre‐intervention trend. Stoklosa 2020 found similar results using a different method and monthly rather than annual data; it found that per capita cigarette sales were increasing at a rate of 0.10 to 0.14 (depending on statistical approach) per month before the introduction of heated tobacco in Japan. After the introduction, per capita cigarette sales declined at a rate of 0.63 to 0.66 cigarettes per month. This study was at moderate risk of bias, due to possible confounding and lack of a preregistered protocol. However, risk of confounding was partially accounted for using regional controls, with the monthly data enabling a sufficient number of time points used to determine pre‐ and postintervention trends across regions.

## Discussion

### Summary of main results

Our searches found no studies that reported the effectiveness of heated tobacco for smoking cessation, but they did find 11 RCTs assessing the safety of heated tobacco — all of which were funded by tobacco companies. Results on adverse and serious adverse events were inconclusive, with insufficient short‐term evidence of differences between smokers randomised to switch to heated tobacco use or to cigarette smoking, attempted tobacco abstinence, or snus use (Table 1; Table 2; Table 3). No studies detected serious harms considered to be related to heated tobacco use. Pooled data showed there was moderate‐certainty evidence that exposure to some measured toxicants and carcinogens was lower in smokers randomised to switch to heated tobacco than continue smoking cigarettes (Table 1), but very low‐ to moderate‐certainty evidence of higher exposures than in those attempting abstinence from all tobacco (Table 2).

No studies directly assessed how trends in smoking prevalence changed following the introduction of heated tobacco to market, but we found two time‐series studies on cigarette sales. Results from both studies showed that the rate of decline in cigarette sales accelerated from before to after the launch of IQOS in Japan (Table 4). However, declining cigarette sales might not translate to falling smoking prevalence, as smokers can reduce the number of cigarettes they smoke without quitting entirely. Moreover, because data were observational, it is possible that changes were caused by other factors (e.g. demographic shifts or delayed effects of tobacco control policies).

### Overall completeness and applicability of evidence

Although included studies had conditions in which they asked smokers to switch completely to HTP or attempt abstinence from all tobacco, none reported smoking cessation outcomes. This means that the effectiveness of heated tobacco for smoking cessation remains uncertain. However, we found one ongoing study that will evaluate their effectiveness relative to e‐cigarettes (Caponnetto 2020).  

Safety data came from a wide range of locations across Europe, Asia, and North America. Conversely, both time‐series studies used data from a single country (Japan), which limits the generalisability of conclusions. For instance, Japan differs from many countries because it is illegal to sell nicotine e‐cigarettes unless they are registered as a pharmaceutical product. This may have left a gap in the market for heated tobacco.

The types of heated tobacco devices produced continues to change over time. While carbon‐tip HTPs such as Eclipse were once the only type available, electronic devices such as IQOS and glo now dominate the market. These products could differ in their safety. It is possible that using newer electronic products, such as those that heat tobacco through induction, could lead to different exposures than those reported here. Therefore, it is important to continue tracking the research into new developments in heated tobacco technology. 

All studies on safety that we included were funded by tobacco companies. These companies have a financial incentive to produce results that are favourable towards the products they sell. Data from independent sources are, therefore, needed to confirm the results reported in this review. We cannot rule out the possibility of publication bias. 

Safety data came from studies that used optimised settings for switching to exclusive HTP use. Six of the 11 RCTs had an extended period where participants stayed in a clinic, preventing those in the HTP group from easily accessing cigarettes (and vice versa). This means that, while trial data consistently show reduced exposure in people completely substituting HTPs for cigarettes, it remains unclear how exposure changes in people using HTPs in real‐world settings where they have greater access to cigarettes.

Serious adverse events were rare as safety data came from studies where participants used heated tobacco for one year at most (median of 13 weeks). Trials with larger samples and longer follow‐up periods are likely needed to establish how switching from cigarettes to heated tobacco affects rates of these events.

Biomarker studies assessing exposure to toxicants and carcinogens are only relevant if reducing exposure prevents disease and premature death. Animal studies have shown a dose–response relationship between some exposures, such as nitrosamines, and cancer development, suggesting reduced exposure may indeed reduce disease incidence (Frank 2007). Nonetheless, longer‐term cohort studies are needed to clarify the impact of switching from cigarettes to heated tobacco. There are several other limitations of biomarker results to consider. First, for biomarkers with an extended half‐life in the body, follow‐up length in some studies may have been too short to accurately estimate the effect of switching from cigarettes to heated tobacco (Goniewicz 2010). Second, all comparisons between heated tobacco and abstinence groups came from RCTs using per‐protocol analyses that excluded people who smoked cigarettes. This exclusion may have introduced selection bias without adequately addressing postrandomisation confounding (Hernán 2017). Finally, we only reported on biomarkers for a sample of the toxicants and carcinogens present in cigarette smoke or heated tobacco aerosol. Previous reviews found similar reductions in exposure to a broader range of potentially harmful chemicals among those switching from cigarettes to heated tobacco (Simonavicius 2018; Znyk 2021).

### Quality of the evidence

We considered the certainty of evidence for effectiveness and safety of heated tobacco compared with cigarette smoking, tobacco abstinence, and snus use, along with population‐level data on smoking prevalence and cigarette sales (Table 1; Table 2; Table 3; Table 4). 

Table 1; Table 2; and Table 3 show evidence from RCTs. Reasons for downgrading certainty of evidence included: risk of bias, when most studies pooled were judged at unclear or high risk of bias; imprecision, when confidence intervals were wide and included no difference; inconsistency, when heterogeneity was high and unexplained; and indirectness, when all the studies pooled used carbon‐tip HTPs, which differ substantially from the electronic devices currently on the market. 

#### Effectiveness

We remain uncertain about the effectiveness of HTPs for smoking cessation, as no studies assessed this.

#### Safety

For all comparisons, effect estimates for adverse events or serious adverse events were of low or very‐low certainty, mainly due to imprecision. This means that we remain uncertain about the direction and size of effects. None of the analyses found serious adverse events that were judged to be caused by HTPs or comparators. For the selected biomarker outcomes NNAL and COHb, evidence was moderate certainty when the comparison was with cigarette smoking; moderate or very‐low certainty compared with tobacco abstinence, respectively; and low or very‐low certainty compared with snus use. This means we are more confident about the effects of heated tobacco on biomarkers relative to cigarettes than to tobacco abstinence or snus.

#### Smoking prevalence

Table 4 shows evidence from time‐series studies investigating smoking prevalence or cigarette sales. We remain uncertain about the impact of rising heated tobacco use on smoking prevalence, as no studies directly assessed this. There was very low‐certainty evidence for an impact on cigarette sales, meaning our confidence in results is limited. We downgraded certainty one level for risk of bias, as the studies were considered at moderate or serious risk of bias. We also downgraded certainty one level for the indirectness of cigarette sales as a proxy for smoking prevalence. This is because falls in cigarette sales do not necessarily translate to reductions in smoking prevalence; people can reduce the number of cigarettes they smoke rather than stopping smoking entirely.

### Potential biases in the review process

We took several steps to ensure the review process was robust. We followed standard methods used by the Cochrane Tobacco Addiction Review Group. Our search strategy included a broad range of databases, including the Cochrane Tobacco Addiction Group Specialised Register. We also contacted researchers who have worked on relevant reports by charities or public health bodies to capture studies that we may have otherwise missed. We followed standard Cochrane practice of requiring two review authors to independently screen studies, extract data, and assess risk of bias. None of the authors of this review were also authors of included studies. 

### Agreements and disagreements with other studies or reviews

Our results were similar to those from an earlier systematic review by Simonavicius 2018, which concluded that HTPs expose "users and bystanders to toxicants, although at substantially lower levels than cigarettes" and noted the lack of studies without links to the tobacco industry. There were analogous results in the Public Health England report into HTPs (McNeill 2018). Our current review differs from these reports because it only uses safety data from RCTs with at least one week of follow‐up. In addition, it includes several studies published between 2018 and 2021 and adds analysis of time‐series studies.

One systematic review by Jankowski 2019 examined data from a wide range of study types, including those using animals and cellular models and those examining the chemical composition of heated tobacco aerosol. Because of these less stringent inclusion criteria, their search identified a greater number of studies than our review (97 versus 16). Nonetheless, they found similar results: "in vitro and in vivo assessments of HTP aerosols revealed reduced toxicity, but these were mainly based on studies sponsored by the tobacco industry". They also reported that exposure to toxicants is likely higher in HTP users compared with those not using any tobacco product. 

One more recent systematic review by Znyk 2021 found that, as we did, there was no evidence on the effectiveness of HTPs for smoking cessation. Their results into the toxicology of HTPs also aligned with ours and with those from the aforementioned reviews.

Finally, prior to the US FDA allowing marketing of IQOS as a "reduced exposure" tobacco product in the US, it reviewed evidence into the safety of these products relative to cigarettes  (FDA 2019). This review concluded that "switching completely from conventional cigarettes to the IQOS system significantly reduces your body's exposure to harmful or potentially harmful chemicals" (FDA 2020). It also emphasised that "the evidence is not sufficient to demonstrate substantiation of either of the claims about reduced risk of tobacco‐related disease or harm". These statements align with our conclusions about the overall completeness of results.

## Authors' conclusions

**Implications for practice**

No studies reported on the use of heated tobacco for cigarette smoking cessation, so their effectiveness for this purpose remains uncertain.  There was insufficient evidence for differences in risk of adverse or serious adverse events between people randomised to use heated tobacco products (HTPs) or to smoke cigarettes, attempt abstinence, or use snus, but participants only used these for a very short time. However, there was moderate‐certainty evidence that users of heated tobacco have lower exposure to selected toxicants and carcinogens than cigarette smokers, and very low‐ to moderate‐certainty evidence of higher exposure than those attempting abstinence from all tobacco. 

The rate of decline in cigarette sales accelerated after the introduction of heated tobacco to market in Japan but, as data were observational, it is possible other factors caused these changes. Moreover, falls in cigarette sales may not translate to declines in smoking prevalence, and changes in Japan may not generalise elsewhere. 

**Implications for research**

Studies from independent sources are needed that attempt to replicate the randomised controlled trials (RCTs) on safety included in this review — all of which were funded by tobacco companies. Users are likely to continue using HTPs for a prolonged period, so studies should allow for this and build in long‐term follow‐up. Studies are also needed to determine how rates of adverse and serious adverse events differ between those randomised to use heated tobacco, continue smoking cigarettes, or use another treatment. Ideally, studies that measure serious adverse events should be powered on this outcome, which is relatively rare, but of key clinical and policy importance. Further studies should measure how biomarkers of exposure and harm differ across groups, especially for new devices. In the longer‐term, large cohort studies and RCTs are needed to examine how long‐term switching from smoking to heated tobacco affects disease incidence and death. If HTPs are determined to be substantially less harmful than traditional cigarettes, RCTs will be needed into their use for cigarette smoking cessation, preferably following up participants for at least six months.

Our literature searches only found population‐level studies examining cigarette sales rather than smoking prevalence. Future research is needed to determine whether the increased rate of decline in cigarette sales following the launch of heated tobacco in Japan translated to similar changes in smoking prevalence trends. Furthermore, to assess whether results generalise, studies need to be conducted in other countries that have also seen substantial growth in heated tobacco use. 

## What's new

**Table:** 

**Date**	**Event**	**Description**
7 April 2022	Amended	Amended to ensure open access status.

## History

Protocol first published: Issue 11, 2020 Review first published: Issue 1, 2022

**Table:** 

**Date**	**Event**	**Description**
4 March 2022	Amended	Analyses 1.4, 1.5 and 1.6 ammended to report MD rather than SMD

## Appendices

### Appendix 1. Biomarkers of toxicant and carcinogen exposure

Where available, we reported on exposure to the following toxicants and carcinogens, using the biomarkers listed below.

- Tobacco‐specific N‐nitrosamine (TSNA) exposure (measured using the biomarker urinary 4‐(methylnitrosamino)‐1‐(3‐pyridyl)‐1‐butanol (NNAL)). Several TSNAs are group 1 or 2A carcinogens, implicated in the increased incidence of cancer among smokers (IARC 2012); NNAL is the most widely investigated biomarker of TSNA exposure (Chang 2017); and NNAL is found in high quantities among cigarette smokers, but very low quantities among NRT and e‐cigarette users (Shahab 2017). Therefore, it also gives an indication of the safety profile of HTPs when compared with other smoking cessation aids.
- Polycyclic aromatic hydrocarbon exposure (measured using the urinary biomarkers 1‐hydroxypyrene (1‐OHP) and 1‐ and 2‐naphthol). Polycyclic aromatic hydrocarbons are produced though incomplete combustion of organic compounds, as occurs through cigarette smoking. Exposure to these compounds is linked to cancers along with DNA, kidney, and liver damage (Kim 2013).
- Exposure to the volatile organic compounds acrolein, heavy metals, and butadiene (measured using the biomarkers 3‐hydroxypropylmercapturic acid (3‐HPMA), heavy metals, and monohydroxy‐3‐butenyl mercapturic acid (MHBMA3), respectively). Acrolein is implicated as the key compound associated with smoking‐induced respiratory disease (Yeager 2016). 3‐hydroxypropylmercapturic acid (3‐HPMA) is a widely used urinary biomarker of acrolein exposure (Schettgen 2008). Carcinogenic heavy metals, like lead and cadmium, are present in cigarette smoke (IARC 2012). Butadiene is a group 1 carcinogen.
- Carbon monoxide (CO) exposure (measured using exhaled CO or carboxyhaemoglobin (COHb) in blood). High exposure to CO among sole heated tobacco products (HTP) users would indicate that the tobacco in HTPs has undergone pyrolysis or combustion. CO exposure is linked to the increased risk of cardiovascular disease among smokers (Hedblad 2005).

### Appendix 2. Data extraction forms

Two custom data extraction forms were produced: one for effectiveness/safety and the other for smoking prevalence. Both included:

- author;
- study design;
- study dates;
- date of publication;
- inclusion and exclusion criteria;
- setting;
- summary of study population characteristics;
- time points at which outcomes were assessed;
- source of study funding;
- author's declarations of interest;
- additional information.

*Effectiveness and safety* forms also included:

- summary of intervention and control conditions, including HTP product and intensity of behavioural support available, where relevant;
- smoking cessation definition used;
- smoking cessation outcomes;
- form of biochemical validation used, where relevant;
- adverse and serious adverse events;
- biomarkers of polycyclic aromatic hydrocarbon exposure (e.g. 1‐OHP) at baseline and longest follow‐up available;
- biomarkers of carbon monoxide exposure (exhaled CO or COHb) at baseline and longest follow‐up point available;
- biomarkers of exposure to the volatile organic compounds including acrolein, lead, cadmium, and butadiene (3‐HPMA, lead, cadmium, and monohydroxy‐3‐butenyl mercapturic acid (MHBMA3), respectively) at baseline and longest follow‐up point available;
- biomarkers of tobacco‐specific N‐nitrosamine (TSNA) exposure (4‐(methylnitrosamino)‐1‐(3‐pyridyl)‐1‐butanol (NNAL)) at baseline and longest follow‐up point available;
- lung function (measured using FEV_1_, FVC and FEV_1_/FVC);
- blood pressure;
- heart rate;
- blood oxygen saturation.

*Smoking prevalence* forms also included:

- coefficient and standard error for change in the trend following intervention in prevalence or sales;
- coefficient and standard error for step‐level change in prevalence or sales;
- coefficient and standard error for changes between cigarette prevalence or sales and heated tobacco product prevalence or sales;
- details of the interruption;
- statistical method used;
- covariates included in model;
- temporal granularity (e.g. weekly, monthly, annual);
- time when heated tobacco products entered the market;
- total time points at which outcomes were assessed;
- pre‐intervention time points;
- postintervention time points;
- stationarity;
- seasonality;
- autocorrelation;
- lags;
- model fit.

### Appendix 3. ROBINS‐I risk of bias for Cummings 2020.

**Table:**

**Domain**	**Judgement**	**Support for judgement**
Bias due to confounding	Serious	Plausible other events could have affected outcomes and no attempt to adjust for such confounding. Limited number (5) of pre‐intervention time points to determine trend.
Bias in selection of participants into the study	Low	Risk of selection bias is unlikely as comprehensive sales data was used.
Bias in classification of interventions	Serious	Joinpoint regression was used to determine pre‐ and postintervention time points, which selects interruption based on outcome data. They also present post‐trend, after 2016 identified as key point, but no analysis of change in that trend.
Bias in deviations from intended interventions	Low	No particular deviations from intended intervention (introduction of heated tobacco to market).
Bias due to missing data	Low	Sales data were reasonably complete.
Bias in measurement of outcomes	Low	Recording of sales data and associated measurement error unrelated to introduction of heated tobacco to market.
Bias in selection of reported result	Moderate	Outcome measures and analyses unregistered, but clearly defined and consistent.

### Appendix 4. ROBINS‐I risk of bias for Stoklosa 2020

**Table:**

**Domain**	**Judgement**	**Support for judgement**
Bias due to confounding	Moderate	Confounding expected, but sufficiently accounted for using regional controls. There were also a sufficient number of time points before and after the intervention to characterise pre‐ and postintervention trends.
Bias in selection of participants into the study	Low	Risk of selection bias is unlikely as sales data from whole regions was used.
Bias in classification of interventions	Low	Interruption time points well defined and not selected based on outcome data.
Bias in deviations from intended interventions	Low	No particular deviations from intended intervention (introduction of heated tobacco to market).
Bias due to missing data	Low	Sales data were reasonably complete.
Bias in measurement of outcomes	Low	Recording of sales data and associated measurement error unrelated to introduction of heated tobacco to market.
Bias in selection of reported result	Moderate	Outcome measures and analyses unregistered, but clearly defined and consistent. Multiple sensitivity analyses reported, but no indication of that these were selected from among multiple analyses.

## Data and analyses

**Comparison 1:** Heated tobacco product (HTP) use compared with cigarette smoking

Outcome or subgroup title	No. of studies	No. of participants	Statistical method	Effect size
1.1 Adverse events	6	1713	Risk Ratio (M‐H, Random, 95% CI)	1.03 [0.92, 1.15]
1.2 Serious adverse events	9	2009	Risk Ratio (M‐H, Random, 95% CI)	0.79 [0.33, 1.94]
1.3 1‐Hydroxypyrene (1‐OHP)	10	1960	Mean Difference (IV, Random, 95% CI)	‐0.42 [‐0.67, ‐0.17]
1.3.1 Intention‐to‐treat	4	1154	Mean Difference (IV, Random, 95% CI)	‐0.58 [‐0.94, ‐0.21]
1.3.2 Per‐protocol	6	806	Mean Difference (IV, Random, 95% CI)	‐0.31 [‐0.68, 0.05]
1.4 1‐Naphthol	1		Mean Difference (IV, Random, 95% CI)	Totals not selected
1.4.1 Per‐protocol	1		Mean Difference (IV, Random, 95% CI)	Totals not selected
1.5 2‐Naphthol	1		Mean Difference (IV, Random, 95% CI)	Totals not selected
1.5.1 Per‐protocol	1		Mean Difference (IV, Random, 95% CI)	Totals not selected
1.6 Exhaled carbon monoxide (CO)	3	1322	Mean Difference (IV, Random, 95% CI)	‐9.13 [‐10.49, ‐7.78]
1.6.1 Intention‐to‐treat	1	858	Mean Difference (IV, Random, 95% CI)	‐6.20 [‐11.01, ‐1.39]
1.6.2 Per‐protocol	2	464	Mean Difference (IV, Random, 95% CI)	‐9.37 [‐10.73, ‐8.01]
1.7 Carboxyhaemoglobin (COHb)	9	1807	Mean Difference (IV, Random, 95% CI)	‐0.74 [‐0.97, ‐0.52]
1.7.1 Intention‐to‐treat	4	1154	Mean Difference (IV, Random, 95% CI)	‐0.92 [‐1.44, ‐0.41]
1.7.2 Per‐protocol	5	653	Mean Difference (IV, Random, 95% CI)	‐0.61 [‐0.82, ‐0.40]
1.8 3‐Hydroxypropylmercapturic acid (3‐HPMA)	10	1960	Mean Difference (IV, Random, 95% CI)	‐0.40 [‐0.62, ‐0.17]
1.8.1 Intention‐to‐treat	4	1154	Mean Difference (IV, Random, 95% CI)	‐0.19 [‐0.28, ‐0.11]
1.8.2 Per‐protocol	6	806	Mean Difference (IV, Random, 95% CI)	‐0.53 [‐0.94, ‐0.13]
1.9 Monohydroxy‐3‐butenyl mercapturic acid (MHBMA)	10	1960	Mean Difference (IV, Random, 95% CI)	‐1.15 [‐1.52, ‐0.78]
1.9.1 Intention‐to‐treat	4	1154	Mean Difference (IV, Random, 95% CI)	‐0.75 [‐1.06, ‐0.43]
1.9.2 Per‐protocol	6	806	Mean Difference (IV, Random, 95% CI)	‐1.41 [‐1.95, ‐0.87]
1.10 4‐(Methylnitrosamino)‐1‐(3‐pyridyl)‐1‐butanol (NNAL)	10	1959	Mean Difference (IV, Random, 95% CI)	‐0.81 [‐1.07, ‐0.55]
1.10.1 Intention‐to‐treat	4	1154	Mean Difference (IV, Random, 95% CI)	‐0.72 [‐1.05, ‐0.38]
1.10.2 Per‐protocol	6	805	Mean Difference (IV, Random, 95% CI)	‐0.89 [‐1.32, ‐0.45]
1.11 Forced expiratory volume in 1 second (FEV_1_)	5	1290	Mean Difference (IV, Random, 95% CI)	0.02 [0.00, 0.03]
1.11.1 Intention‐to‐treat	1	858	Mean Difference (IV, Random, 95% CI)	0.01 [0.00, 0.03]
1.11.2 Per‐protocol	4	432	Mean Difference (IV, Random, 95% CI)	0.02 [‐0.00, 0.04]
1.12 Systolic blood pressure	3	288	Mean Difference (IV, Random, 95% CI)	0.00 [‐0.02, 0.02]
1.12.1 Per‐protocol	3	288	Mean Difference (IV, Random, 95% CI)	0.00 [‐0.02, 0.02]
1.13 Diastolic blood pressure	3	288	Mean Difference (IV, Random, 95% CI)	‐0.00 [‐0.03, 0.03]
1.13.1 Per‐protocol	3	288	Mean Difference (IV, Random, 95% CI)	‐0.00 [‐0.03, 0.03]
1.14 Forced vital capacity (FVC)	2	196	Mean Difference (IV, Random, 95% CI)	‐0.12 [‐0.45, 0.21]
1.14.1 Per‐protocol	2	196	Mean Difference (IV, Random, 95% CI)	‐0.12 [‐0.45, 0.21]

**Comparison 2:** Heated tobacco product (HTP) use compared with abstinence from tobacco

Outcome or subgroup title	No. of studies	No. of participants	Statistical method	Effect size
2.1 Adverse events	2	237	Risk Ratio (M‐H, Random, 95% CI)	1.12 [0.86, 1.46]
2.2 Serious adverse events	5	533	Risk Ratio (M‐H, Random, 95% CI)	Not estimable
2.3 1‐Hydroxypyrene (1‐OHP)	5	382	Mean Difference (IV, Random, 95% CI)	0.12 [‐0.03, 0.28]
2.3.1 Intention‐to‐treat	3	212	Mean Difference (IV, Random, 95% CI)	0.11 [‐0.03, 0.25]
2.3.2 Per‐protocol	2	170	Mean Difference (IV, Random, 95% CI)	0.22 [‐0.32, 0.75]
2.4 Carboxyhaemoglobin (COHb)	5		Mean Difference (IV, Random, 95% CI)	Subtotals only
2.4.1 Intention‐to‐treat	3	212	Mean Difference (IV, Random, 95% CI)	0.69 [0.07, 1.31]
2.4.2 Per‐protocol	2	170	Mean Difference (IV, Random, 95% CI)	‐0.32 [‐1.04, 0.39]
2.5 3‐Hydroxypropylmercapturic acid (3‐HPMA)	5	382	Mean Difference (IV, Random, 95% CI)	0.56 [0.33, 0.80]
2.5.1 Intention‐to‐treat	3	212	Mean Difference (IV, Random, 95% CI)	0.64 [0.32, 0.96]
2.5.2 Per‐protocol	2	170	Mean Difference (IV, Random, 95% CI)	0.35 [0.20, 0.50]
2.6 Monohydroxy‐3‐butenyl mercapturic acid (MHBMA)	5	382	Mean Difference (IV, Random, 95% CI)	0.67 [‐0.12, 1.45]
2.6.1 Intention‐to‐treat	3	212	Mean Difference (IV, Random, 95% CI)	0.97 [0.02, 1.92]
2.6.2 Per‐protocol	2	170	Mean Difference (IV, Random, 95% CI)	0.07 [‐0.16, 0.30]
2.7 4‐(Methylnitrosamino)‐1‐(3‐pyridyl)‐1‐butanol (NNAL)	5	382	Mean Difference (IV, Random, 95% CI)	0.50 [0.34, 0.66]
2.7.1 Intention‐to‐treat	3	212	Mean Difference (IV, Random, 95% CI)	0.51 [0.34, 0.69]
2.7.2 Per‐protocol	2	170	Mean Difference (IV, Random, 95% CI)	0.42 [‐0.01, 0.85]
2.8 Forced expiratory volume in 1 second (FEV_1_)	2	170	Mean Difference (IV, Random, 95% CI)	‐0.00 [‐0.06, 0.06]
2.8.1 Per‐protocol	2	170	Mean Difference (IV, Random, 95% CI)	‐0.00 [‐0.06, 0.06]
2.9 Systolic blood pressure	2	170	Mean Difference (IV, Random, 95% CI)	0.02 [‐0.01, 0.05]
2.9.1 Per‐protocol	2	170	Mean Difference (IV, Random, 95% CI)	0.02 [‐0.01, 0.05]
2.10 Diastolic blood pressure	2	170	Mean Difference (IV, Random, 95% CI)	0.00 [‐0.04, 0.04]
2.10.1 Per‐protocol	2	170	Mean Difference (IV, Random, 95% CI)	0.00 [‐0.04, 0.04]
2.11 Forced vital capacity (FVC)	2	172	Mean Difference (IV, Random, 95% CI)	‐0.02 [‐0.29, 0.26]
2.11.1 Per‐protocol	2	172	Mean Difference (IV, Random, 95% CI)	‐0.02 [‐0.29, 0.26]

**Comparison 3:** Heated tobacco product (HTP) use compared with snus use

Outcome or subgroup title	No. of studies	No. of participants	Statistical method	Effect size
3.1 Adverse events	1		Risk Ratio (M‐H, Random, 95% CI)	Totals not selected
3.2 Serious adverse events	1		Risk Ratio (M‐H, Random, 95% CI)	Totals not selected
3.3 1‐Hydroxypyrene (1‐OHP)	1		Mean Difference (IV, Random, 95% CI)	Totals not selected
3.4 1‐Naphthol	1		Mean Difference (IV, Random, 95% CI)	Totals not selected
3.5 2‐Naphthol	1		Mean Difference (IV, Random, 95% CI)	Totals not selected
3.6 Carboxyhaemoglobin (COHb)	1		Mean Difference (IV, Random, 95% CI)	Totals not selected
3.7 3‐Hydroxypropylmercapturic acid (3‐HPMA)	1		Mean Difference (IV, Random, 95% CI)	Totals not selected
3.8 Monohydroxy‐3‐butenyl mercapturic acid (MHBMA)	1		Mean Difference (IV, Random, 95% CI)	Totals not selected
3.9 4‐(Methylnitrosamino)‐1‐(3‐pyridyl)‐1‐butanol (NNAL)	1		Mean Difference (IV, Random, 95% CI)	Totals not selected

## Characteristics of studies

### Characteristics of included studies [ordered by study ID]

**Table:** Bosilkovska 2020

***Study characteristics***		
Methods	Design: RCT Study dates: January 2016 to August 2016 Recruitment: from clinic's database and advertisements Location: Warsaw, Poland Setting: in a clinic for 5 days then an ambulatory setting for following 85 days	
Participants	Number randomised: 120 Characteristics: 46.7% women; mean age 38.9 years; 45.0% smoked 10–19 cigarettes per day, while 55.0% smoked > 19 cigarettes per day; mean FTND score 5.3 Specialist population: aged ≥ 28 years; Caucasian Inclusion criteria Healthy Verified current smoker (≥ 10 non‐menthol cigarettes per day for past 6 weeks) Aged ≥ 28 years Caucasian origin Smoked for previous ≥ 10 years No intention to quit smoking in the next 6 months Exclusion criteria Medical conditions that were a safety concern or would interfere with study BMI < 18.5 or ≥ 32 kg/m^2^ Use of nicotine‐containing products other than cigarettes in 6 weeks prior to admission Use of drugs likely to affect CYP1A2 or CYP2A6 activity within 14 days or 5 half‐lives of the drug (whichever was longer) before admission Current or past alcohol problems Positive urine drug test Given or received blood in 3 months prior to admission Current or past employee of the tobacco industry or their close relatives Pregnant or breastfeeding People of childbearing potential who do not agree to use contraception	
Interventions	Randomised (2:1 ratio) to use a carbon tip HTP or continue smoking cigarettes **Heated tobacco arm** Device heating method: carbon tip Device name: CHTP 1.2 Device manufacturer: PMI Other instructions and details: CHTP products were provided to participants randomised to heated tobacco arm Behavioural support: record use of nicotine and tobacco products in electronic diary. 5 days in clinic setting Instructions for smoking cessation/switching: only use CHTP HTP for study period **Cigarette smoking arm** Behavioural support: record use of nicotine and tobacco products in electronic diary. 5 days in clinic setting Instructions for smoking cessation/switching: continue smoking cigarettes	
Outcomes	Follow‐up time points: 13 weeks Abstinence outcomes: N/A Safety outcomes: biomarkers of toxicant and carcinogen exposure; biomarkers of harm; adverse events; serious adverse events	
Notes	**Funding source** Tobacco industry funded: quote: "Philip Morris International is the sole source of funding and the sponsor of this study". **Author conflicts of interest** Quote: "All authors are employees of Philip Morris International (PMI) or work for PMI under contractual agreements".	
***Risk of bias***		
**Bias**	**Authors' judgement**	**Support for judgement**
Random sequence generation (selection bias)	Unclear risk	No details on random sequence generation.
Allocation concealment (selection bias)	Low risk	Protocol mentions that: "At the end of the baseline period enrolled subjects will be randomized using an interactive web and voice response system (IxRS) on day −1 at any time during the day".
Blinding of outcome assessment (detection bias) All outcomes	Low risk	All outcomes were biological and assessors were blinded to randomised group allocation.
Incomplete outcome data (attrition bias) All outcomes	Low risk	Low attrition in both groups, 76/80 participants in heated tobacco arm and 39/40 in cigarette arm completed final follow‐up.
Selective reporting (reporting bias)	Low risk	All preregistered outcomes reported.

**Table:** Cummings 2020

***Study characteristics***		
Methods	Design: interrupted time‐series Study dates: 2011–2019 Data source: sales data from the Tobacco Institute of Japan and PMI Location: Japan	
Participants	Heated tobacco use prevalence assessed? no Definition of heated tobacco use prevalence: N/A Participant characteristics: N/A Heated tobacco sales assessed? yes Definition of heated tobacco sales: billions of heated tobacco sticks sold. 1 pack of Ploom Tech consumables was assumed equivalent to 20 combustible cigarettes	
Interventions	Interruption time point: 2016 Method used to select interruption: interruption time point was not prespecified, but instead selected from the data using Joinpoint regression Other details: trend analyses performed in Joinpoint 4.7, which produces a segmented regression where the number of breakpoints are selected based on statistical significance	
Outcomes	Abstinence outcomes: N/A Safety outcomes: N/A Prevalence/sales outcomes: difference in yearly percentage reduction in cigarette sales Definition of smoking prevalence: N/A Definition of cigarette sales: billions of cigarette sticks sold	
Notes	**Funding source** No funding from the tobacco industry: quote: "K.M.C. and G.J.N. receive funding support from grants from the US National Cancer Institute (P01 CA200512, P30 CA138313)". **Author conflicts of interest** Quote: "K.M.C. has been a consultant and received grant funding from Pfizer, Inc. in the past five years. K.M.C. has also been a paid expert witness in litigation against the cigarette industry. D.T.S. does not accept money from any entity with a financial interest in promoting any tobacco or nicotine product, nor from any organization that promotes an abstinence‐only position on nicotine and tobacco products".

**Table:** Gale 2020

***Study characteristics***		
Methods	Design: RCT Study dates: February 2018 to March 2020 Recruitment: not reported Location: UK (Leeds, Belfast, London, and Merthyr Tydfil) Setting: ambulatory setting	
Participants	Number randomised: 276 Characteristics: only reported demographics from per‐protocol population. 46.8% women; mean age 38–39 years; mean 18 cigarettes smoked per day; mean FTND score 5–6 Specialist population: aged 28–55 years Inclusion criteria Healthy Smoked cigarettes for previous ≥ 5 years Aged 28–55 years Agreed to refrain from alcohol for 24 hours before study visits No intention to quit smoking Exclusion criteria Medical conditions that were a safety concern or would interfere with study BMI < 17.6 or ≥ 32.0 kg/m^2^ Bodyweight < 50 kg for men or < 40 kg for women Use of nicotine‐containing products other than cigarettes in 14 days prior to screening Use of drugs likely to interfere with study Current or past alcohol or drug problems Self‐report not inhaling smoke from cigarettes into lungs Strenuous exercise 7 days prior to screening Pregnant or breastfeeding People of childbearing potential who do not agree to use contraception	
Interventions	Randomised (2.5:1 ratio) to use an electronic HTP or continue smoking cigarettes **Heated tobacco arm** Device heating method: electronic Device name: glo Device manufacturer: BAT Other instructions and details: provided with tobacco sticks equivalent to 150% their mean number of cigarettes smoked per day initially, and 120% of their use in the previous period Behavioural support: record use of nicotine and tobacco products in electronic diary. Instructed on importance of exclusively using HTP Instructions for smoking cessation/switching: only use glo HTP for 12 months **Cigarette smoking arm** Behavioural support: record use of nicotine and tobacco products in electronic diary Instructions for smoking cessation/switching: continue smoking cigarettes for study period	
Outcomes	Follow‐up time points: 4, 9, 13, 26, and 52 weeks Abstinence outcomes: N/A Safety outcomes: biomarkers of toxicant and carcinogen exposure; biomarkers of harm; adverse events; serious adverse events Prevalence/sales outcomes: N/A	
Notes	**Funding source** Tobacco industry funded: quote: "The study was supported by British American Tobacco (Investments) Limited". **Author conflicts of interest** Quote: "All authors are current employees of British American Tobacco (Investments) Limited".	
***Risk of bias***		
**Bias**	**Authors' judgement**	**Support for judgement**
Random sequence generation (selection bias)	Low risk	Randomised using (quote): "blocks of computer generated random number sequences in PROC PLAN".
Allocation concealment (selection bias)	Unclear risk	No details on whether random sequence was concealed from investigators.
Blinding of outcome assessment (detection bias) All outcomes	Low risk	All outcomes were biological.
Incomplete outcome data (attrition bias) All outcomes	Low risk	Similar attrition in both groups, with 127/197 participants in heated tobacco arm and 59/79 in cigarette arm completing the final follow‐up.
Selective reporting (reporting bias)	Low risk	All preregistered outcomes reported.

**Table:** Haziza 2019

***Study characteristics***		
Methods	Design: RCT Study dates: December 2013 to October 2014 Recruitment: not reported Location: USA (Texas, Florida) Setting: 5 days in clinic, and 86 days in an ambulatory setting	
Participants	Number randomised: 147 (excluding 13 who were misrandomised) Characteristics: 40.0% women; mean age 37.7 years; 51.3% smoked 10–19 cigarettes per day, 48.1% smoked > 19 cigarettes per day, and 0.6% had missing data; mean FTND score 5.6 Specialist population: aged ≥ 22 years; menthol cigarette smokers Inclusion criteria Healthy Currently smoke ≥ 10 menthol cigarettes per day, verified using urinary cotinine Smoked menthol cigarettes for past ≥ 3 years Aged ≥ 22 years Agree to refrain from alcohol for 24 hours before study visits No intention to quit smoking within next 6 months Exclusion criteria Medical conditions that were a safety concern or would interfere with study BMI < 18.5 or ≥ 35.0 kg/m^2^ Use of nicotine‐containing products other than menthol cigarettes in 4 weeks prior to screening Use of drugs likely to interfere with study Current or past alcohol or drug problems Gave or received blood in past 3 months Pregnant or breastfeeding People of childbearing potential who did not agree to use contraception Current or past employee of tobacco industry or their close relatives	
Interventions	Randomised (2:1:1 ratio) to use an electronic HTP, continue smoking cigarettes, or become abstinent **Heated tobacco arm** Device heating method: electronic Device name: menthol THS 2.2, brand name IQOS Device manufacturer: PMI Other instructions and details: provided with menthol tobacco sticks Behavioural support: 5 days in a clinic setting, where product use was monitored. Record use of nicotine and tobacco products in electronic diary. Carbon monoxide breath tests were used to assess compliance Instructions for smoking cessation/switching: switch entirely to menthol THS 2.2 for 90‐day study period **Cigarette smoking arm** Behavioural support: record use of nicotine and tobacco products in electronic diary Instructions for smoking cessation/switching: not advised to stop smoking **Abstinence arm** Behavioural support: record use of nicotine and tobacco products in electronic diary. Carbon monoxide breath tests used to assess compliance Instructions for smoking cessation/switching: do not smoke cigarettes during 90‐day study period	
Outcomes	Follow‐up time points: 4, 9, and 19 weeks Abstinence outcomes: N/A Safety outcomes: biomarkers of toxicant and carcinogen exposure; biomarkers of harm; adverse events; serious adverse events Prevalence/sales outcomes: N/A	
Notes	**Funding source** Tobacco industry funded: quote: "Philip Morris International is the sole source of funding and sponsor of this project". **Author conflicts of interest** Quote: "All authors are employees of Philip Morris International".	
***Risk of bias***		
**Bias**	**Authors' judgement**	**Support for judgement**
Random sequence generation (selection bias)	Unclear risk	No details on random sequence generation.
Allocation concealment (selection bias)	Low risk	Randomised using an interactive voice response system.
Blinding of outcome assessment (detection bias) All outcomes	Low risk	All outcomes were biological.
Incomplete outcome data (attrition bias) All outcomes	High risk	Attrition differed substantially across groups, with 59% participants in heated tobacco arm, 78% in cigarette arm, and 23% in abstinent arm completing final 90‐day follow‐up — with others removed due to protocol violations, non‐compliance, or discontinuation.
Selective reporting (reporting bias)	Low risk	All preregistered outcomes reported.
Other bias	Unclear risk	Reported that there were more people in the 90‐day follow‐up sample than at baseline.

**Table:** Lüdicke 2018

***Study characteristics***		
Methods	Design: RCT Study dates: August 2013 to July 2014 Recruitment: clinical database and advertisements Location: Tokyo, Japan Setting: 5 days in clinic, and 85 days in an ambulatory setting	
Participants	Number randomised: 160 Characteristics: 42.5% women; mean age 37.2 years; 52.5% smoked 10–19 cigarettes per day, 47.5% smoked > 19 cigarettes per day; mean FTND score of 4.4 Specialist population: aged 22–65 years; Japanese; menthol cigarette smoker Inclusion criteria Healthy Currently smoke ≥ 10 menthol cigarettes per day Smoked menthol cigarettes for at past ≥ 3 years Aged 22–65 years No plan to quit smoking within next 3 months Exclusion criteria Medical conditions that were a safety concern or would interfere with study BMI < 18.5 or > 32.0 kg/m^2^ Use of nicotine‐containing products other than menthol cigarettes in 4 weeks prior to screening Use drugs likely to interfere with study Current or past alcohol problems Positive urine drug test Gave or received blood in past 3 months Pregnant or breastfeeding People of childbearing potential who do not agree to use contraception Current or past employee of tobacco industry (or their close relatives)	
Interventions	Randomised (2:1:1 ratio) to use an electronic HTP, continue smoking cigarettes, or become abstinent **Heated tobacco arm** Device heating method: electronic Device name: menthol THS 2.2, brand name IQOS Device manufacturer: PMI Other instructions and details: provided with menthol tobacco sticks Behavioural support: 5 days in a clinic setting, where product use was monitored. Record use of nicotine and tobacco products in electronic diary. Carbon monoxide breath tests were used to assess compliance Instructions for smoking cessation/switching: switch entirely to menthol THS 2.2 for study period **Cigarette smoking arm** Behavioural support: record use of nicotine and tobacco products in electronic diary Instructions for smoking cessation/switching: not advised to stop smoking **Abstinence arm** Behavioural support: record use of nicotine and tobacco products in electronic diary. Carbon monoxide breath tests used to assess compliance. Nicotine replacement therapy was allowed, but not provided Instructions for smoking cessation/switching: do not smoke cigarettes during study period	
Outcomes	Follow‐up time points: 4, 9, and 13 weeks Abstinence outcomes: N/A Safety outcomes: biomarkers of toxicant and carcinogen exposure; biomarkers of harm; adverse events; serious adverse events Prevalence/sales outcomes: N/A	
Notes	**Funding source** Tobacco industry funded: quote: "The study was funded by Philip Morris Products S.A.". **Author conflicts of interest** Quote: "All authors are employees of Philip Morris Products S.A.".	
***Risk of bias***		
**Bias**	**Authors' judgement**	**Support for judgement**
Random sequence generation (selection bias)	Unclear risk	No details on random sequence generation.
Allocation concealment (selection bias)	Unclear risk	Randomised was performed using an interactive voice response system.
Blinding of outcome assessment (detection bias) All outcomes	Low risk	All outcomes were biological.
Incomplete outcome data (attrition bias) All outcomes	Low risk	Low attrition reported across all groups, with 97% of participants who were randomised the heated tobacco, 98% to cigarette smoking, and 95% to abstinence arms completing the final follow‐up.
Selective reporting (reporting bias)	High risk	Did not report FVC outcomes, which were preregistered.

**Table:** Lüdicke 2019

***Study characteristics***		
Methods	Design: RCT Study dates: March 2015 to December 2016 Recruitment: from an existing database of study volunteers and local advertising Location: USA (Arizona, Florida, Kentucky, Nebraska, Nevada, North Carolina, Ohio, Tennessee, Texas, and Virginia) Setting: ambulatory setting	
Participants	Number randomised: 984 Characteristics: demographics only reported for complete cases. 41.2% women; mean age 44.6 years; mean cigarettes per day 19.3; mean FTND score 5.8 Specialist population: aged ≥ 30 years Inclusion criteria Healthy Verified current smoker Aged ≥ 30 years Smoked for the last 10 years Smoked > 10 commercially available non‐menthol cigarettes per day on average over past year Not motivated to quit smoking within next 6 months Exclusion criteria Medical conditions that investigators judged to be safety concern FEV_1_/FVC < 0.7 and FEV_1_ < 80% predicted value at postbronchodilator spirometry Asthma BMI <18.5 or ≥ 35 kg/m^2^ Taking medication which may impact on the smoker's health profile Pregnant or breastfeeding People of childbearing potential who did not agree to use contraception	
Interventions	Randomised (2:1 ratio) to use an electronic HTP or continue smoking cigarettes **Heated tobacco arm** Device heating method: electronic Device name: THS 2.2, brand name IQOS Device manufacturer: PMI Other instructions and details: received training on using THS 2.2. Given tobacco sticks (HeatSticks) to cover needs until next visit Behavioural support: asked to record all tobacco/nicotine product use in an electronic diary Instructions for smoking cessation/switching: use HTP exclusively for 6 months **Cigarette smoking arm** Behavioural support: asked to record all tobacco/nicotine product use in an electronic diary Instructions for smoking cessation/switching: continue smoking own brand of cigarettes	
Outcomes	Follow‐up time points: 26 and 52 weeks Abstinence outcomes: N/A Safety outcomes: biomarkers of toxicant and carcinogen exposure; biomarkers of harm; adverse events; serious adverse events Prevalence/sales outcomes: N/A	
Notes	**Funding source** Tobacco industry funded: Quote: "PMI is the sole source of funding and sponsor of this project". **Author conflicts of interest** Quote: "S.M. Ansari is a Clinical Scientist at Philip Morris International. N. Lama is a Senior Scientist – Statistics at Philip Morris International. P. Picavet has ownership interest (including stock, patents, etc.) in Philip Morris International. G. Baker has ownership interest (including stock, patents, etc.) in Philip Morris International. M. Peitsch has ownership interest (including stock, patents, etc.) in Philip Morris International. R. Weitkunat has ownership interest (including stock, patents, etc.) in Philip Morris International. No potential conflicts of interest were disclosed by the other authors".	
***Risk of bias***		
**Bias**	**Authors' judgement**	**Support for judgement**
Random sequence generation (selection bias)	Unclear risk	No details on random sequence generation.
Allocation concealment (selection bias)	Low risk	Randomised using an interactive voice response system.
Blinding of outcome assessment (detection bias) All outcomes	Low risk	All outcomes were biological.
Incomplete outcome data (attrition bias) All outcomes	Low risk	Low attrition in both groups (15.1% in heated tobacco arm, 10.5% in cigarette smoking arm).
Selective reporting (reporting bias)	Low risk	All preregistered primary outcomes reported.

**Table:** Martin 2012

***Study characteristics***		
Methods	Design: RCT Study dates: October 2007 to April 2008 Recruitment: clinical database Location: Warsaw, Poland Setting: ambulatory setting, with weekly visits to clinic	
Participants	N randomised: 316 Characteristics: 49% women; mean age 43.6 years; mean 25 cigarettes per day; mean FTND score 5.9 Specialist population: aged 30–60 years; Caucasian ethnicity Inclusion criteria Currently smoke at least non‐menthol cigarettes Smoked non‐menthol cigarettes for past ≥ 10 years Aged 30–60 years Exclusion criteria Unacceptable health conditions Clinically relevant abnormal findings at screening Pregnant or breastfeeding People of childbearing potential who did not agree to use contraception	
Interventions	Randomised (3:1 ratio) to use an electronic HTP or continue smoking cigarettes **Heated tobacco arm** Device heating method: electronic Device name: EHCSS‐K6, pre‐cursor to THS marketed under brand name IQOS Device manufacturer: PMI Other instructions and details: provided with tobacco sticks Behavioural support: trained to use HTP. Asked to record use of nicotine and tobacco products in electronic diary to assess compliance Instructions for smoking cessation/switching: switch entirely to HTP provided for study period **Cigarette smoking arm** Behavioural support: record use of nicotine and tobacco products in electronic diary Instructions for smoking cessation/switching: continue smoking conventional cigarettes	
Outcomes	Follow‐up time points: 4 and 5 weeks Abstinence outcomes: N/A Safety outcomes: biomarkers of toxicant and carcinogen exposure; biomarkers of harm; adverse events; serious adverse events Prevalence/sales outcomes: N/A	
Notes	**Funding source** Tobacco industry funded: quote: "The work reported in all eight parts of this supplement was funded by PMI R&D". **Author conflicts of interest** Quote: "All authors are or were Philip Morris International (PMI) R&D employees or worked for PMI R&D under contractual agreements".	
***Risk of bias***		
**Bias**	**Authors' judgement**	**Support for judgement**
Random sequence generation (selection bias)	Unclear risk	No details on random sequence generation.
Allocation concealment (selection bias)	Low risk	Randomised using an Interactive Voice Response System.
Blinding of outcome assessment (detection bias) All outcomes	Low risk	All outcomes were biological.
Incomplete outcome data (attrition bias) All outcomes	Low risk	Low attrition across both groups, with 99% of participants randomised to heated tobacco use and 95% to cigarette smoking remaining in the study until the final follow‐up.
Selective reporting (reporting bias)	Unclear risk	No protocol or analysis plan was registered online.

**Table:** NCT03364751

***Study characteristics***		
Methods	Design: RCT Study dates: November 2017 to June 2019 Recruitment: recruited at dental practices Location: Japan Setting: ambulatory setting, with data collected at dental clinics	
Participants	Number randomised: 172 Characteristics: 19.2% women; mean age 48 years; all smoked > 10 cigarettes per day; no data on FTND score Specialist population: aged ≥ 30 years; Japanese ethnicity; diagnosed with generalised chronic periodontitis Inclusion criteria Current smoker, verified by urinary cotinine Smoked ≥ 10 cigarettes per day for past ≥ 5 years Aged ≥ 30 years Diagnosed with generalised chronic periodontitis Had ≥ 15 natural teeth No intention to quit smoking during study Exclusion criteria Medical conditions that were a safety concern or would interfere with study Had orthodontic appliances Use drugs or supplements likely to interfere with study Pregnant or lactating Planning pregnancy during study period	
Interventions	Randomised (1:1 ratio) to use an electronic HTP or continue smoking cigarettes **Heated tobacco arm** Device heating method: electronic Device name: THS, brand name IQOS Device manufacturer: PMI Other instructions and details: participants were given THS devices, but asked to buy their own tobacco sticks Behavioural support: none mentioned Instructions for smoking cessation/switching: switch entirely to THS use for study period **Cigarette smoking arm** Behavioural support: none mentioned Instructions for smoking cessation/switching: continue smoking cigarettes	
Outcomes	Follow‐up time points: 13 and 26 weeks Abstinence outcomes: N/A Safety outcomes: adverse events; serious adverse events Prevalence/sales outcomes: N/A	
Notes	**Funding source** Tobacco industry funded: quote: "PMI is the sole source of funding and sponsor of this project". **Author conflicts of interest** Quote: "The work reported in this publication involved a candidate reduced risk product developed by PMI Research & Development. All authors are employees of PMI".	
***Risk of bias***		
**Bias**	**Authors' judgement**	**Support for judgement**
Random sequence generation (selection bias)	Unclear risk	No details on random sequence generation.
Allocation concealment (selection bias)	Low risk	Randomised using an interactive web/voice response system.
Blinding of outcome assessment (detection bias) All outcomes	Low risk	All outcomes were biological.
Incomplete outcome data (attrition bias) All outcomes	Low risk	Attrition was low across all groups, with only 1 participant failing to complete follow‐up in each study arm.
Selective reporting (reporting bias)	Low risk	Protocol was preregistered and all outcomes were reported.
Other bias	High risk	Results analysed based on actual product use, rather than product participants were randomly allocated to use.

**Table:** Ogden 2015

***Study characteristics***		
Methods	Design: RCT Study dates: November 2007 to November 2009 Recruitment: no details Location: USA (Idaho, Texas, Florida, and Oregon) Setting: ambulatory setting, with in 24‐hour of confinement in‐clinic at weeks 0, 12, and 24	
Participants	Number randomised: 131 Characteristics: 49.6% women; mean age 42 years; no information on mean cigarettes smoked per day or FTND score Specialist population: aged 28–55 years Inclusion criteria Currently smoke ≥ 15 cigarettes per day Smoked cigarettes for past ≥ 10 years Aged 28–55 years Agree to refrain from alcohol for 24 hours before study visits No intention to quit smoking within next month, but willing to switch to alternative tobacco product Exclusion criteria Medical conditions that were a safety concern or would interfere with study BMI < 18.5 or ≥ 35.0 kg/m^2^ Use of nicotine‐containing products other than cigarettes in 6 months prior to screening Use drugs or supplements likely to interfere with study Positive alcohol or drug test Past alcohol or drug problems Given blood in past 30 days, or received blood in past 2 months Pregnant or lactating People of childbearing potential who did not agree to use contraception	
Interventions	Randomised (1:1:1 ratio) to use a carbon tip HTP, snus, or continue smoking cigarettes **Heated tobacco arm** Device heating method: carbon tip Device name: Eclipse Device manufacturer: R.J Reynolds (BAT) Other instructions and details: provided with heated tobacco sticks, with choice of menthol or non‐menthol products Behavioural support: provided information about HTP. Compensated for recording use of nicotine and tobacco products in interactive voice recording system diary Instructions for smoking cessation/switching: switch entirely to Eclipse for study period **Snus arm** Other instructions and details: provided snus with 3 flavour options (spice, original, and frost) Behavioural support: provided information about snus. Compensated for recording use of nicotine and tobacco products in interactive voice response system diary Instructions for smoking cessation/switching: switch to snus use for study period **Cigarette smoking arm** Other instructions and details: provided with ultra‐low machine yield cigarettes, with choice of menthol or non‐menthol cigarettes Behavioural support: compensated for recording use of nicotine and tobacco products in interactive voice recording system diary Instructions for smoking cessation/switching: switch to smoking ultra‐low machine yield cigarettes	
Outcomes	Follow‐up time points: 12 and 24 weeks Abstinence outcomes: N/A Safety outcomes: biomarkers of toxicant and carcinogen exposure; adverse events; serious adverse events Prevalence/sales outcomes: N/A	
Notes	**Funding source** Tobacco industry funded: quote: "All authors are current employees of RAI Services Company or R.J. Reynolds Tobacco Company". **Author conflicts of interest** Quote: "All authors are current employees of RAI Services Company or R.J. Reynolds Tobacco Company".	
***Risk of bias***		
**Bias**	**Authors' judgement**	**Support for judgement**
Random sequence generation (selection bias)	Unclear risk	No details on random sequence generation.
Allocation concealment (selection bias)	Low risk	Randomised using an Interactive Voice Response System.
Blinding of outcome assessment (detection bias) All outcomes	Low risk	All outcomes were biological.
Incomplete outcome data (attrition bias) All outcomes	Low risk	Attrition 25% overall, but differed by < 20% points across groups (23% in heated tobacco, 33% in snus, and 21% in cigarette arms).
Selective reporting (reporting bias)	Unclear risk	No preregistered analysis plan, as trial registration was added several years after data were collected.

**Table:** Stoklosa 2020

***Study characteristics***		
Methods	Design: interrupted time‐series Study dates: 2014–2018 Data source: sales data from Intage Inc., a market research company that collects monthly sales data from supermarkets and convenience stores Location: Japan	
Participants	Heated tobacco use prevalence assessed? No Definition of heated tobacco use prevalence: N/A Participant characteristics: N/A Heated tobacco sales assessed? Yes Definition of heated tobacco sales: heated tobacco sticks sold per capita	
Interventions	Interruption time point: September 2015 or April 2016 Method used to select interruption: interruption time point was selected depending on when IQOS was introduced to market in each prefecture	
Outcomes	Abstinence outcomes: N/A Safety outcomes: N/A Prevalence/sales outcomes: difference in monthly reduction in cigarette sales per capita Definition of smoking prevalence: N/A Definition of cigarette sales: cigarette sticks sold per capita	
Notes	**Funding source** No funding from the tobacco industry: quote: "This research received no specific grant from any funding agency in the public, commercial or not‐for‐profit sectors". **Author conflicts of interest** Quote: "None declared".

**Table:** Tricker 2012a

***Study characteristics***		
Methods	Design: RCT Study dates: not reported Recruitment: no information Location: Seoul, South Korea Setting: confinement in‐clinic setting	
Participants	Number randomised: 72 Characteristics: 25.0% women; mean age 23.8 years; all participants smoked ≥ 10 cigarettes per day; mean FTND score 3.9 for men and 3.3 for women Specialist population: aged 20–50 years Inclusion criteria Currently smoke 10–30 cigarettes per day Aged 20–50 years Only smoked Lark1 cigarettes for 2 weeks prior to study Exclusion criteria Unacceptable medical conditions Abnormal findings on physical examination Use of nicotine or tobacco product other than cigarettes within 3 months prior to screening Alcohol or drug problems Use of any medication other than hormonal contraceptives Pregnant or lactating People of childbearing potential who did not agree to use contraception	
Interventions	Randomised (2:2:1 ratio) to use an electronic HTP, continue smoking cigarettes, or become abstinent **Heated tobacco arm** Device heating method: electronic Device name: EHCSS‐K3, precursor to THS marketed under brand name IQOS Device manufacturer: PMI Other instructions and details: participants were given tobacco sticks Behavioural support: none mentioned Instructions for smoking cessation/switching: switch entirely to heated tobacco use for study period **Cigarette smoking arm** Other instructions and details: participants were given Lark1 low yield cigarettes Behavioural support: none mentioned Instructions for smoking cessation/switching: continue smoking Lark1 cigarettes for study period **Abstinence arm** Behavioural support: none mentioned Instructions for smoking cessation/switching: do not smoke cigarettes during study period	
Outcomes	Follow‐up time points: 1 week (8 days) Abstinence outcomes: N/A Safety outcomes: biomarkers of exposure to toxins and carcinogens; biomarkers of harm; adverse events; serious adverse events Prevalence/sales outcomes: N/A	
Notes	**Funding source** Tobacco industry funded: quote: "The work reported in all eight parts of this supplement was funded by PMI R&D". **Author conflicts of interest** Quote: "All authors are or were Philip Morris International (PMI) R&D employees or worked for PMI R&D under contractual agreements".	
***Risk of bias***		
**Bias**	**Authors' judgement**	**Support for judgement**
Random sequence generation (selection bias)	Unclear risk	No details on random sequence generation.
Allocation concealment (selection bias)	Unclear risk	No information.
Blinding of outcome assessment (detection bias) All outcomes	Low risk	All outcomes were biological.
Incomplete outcome data (attrition bias) All outcomes	Unclear risk	No information on attrition.
Selective reporting (reporting bias)	Unclear risk	No trial registration or analysis plan.

**Table:** Tricker 2012b

***Study characteristics***		
Methods	Design: RCT Study dates: not reported Recruitment: no information Location: Japan Setting: confinement in‐clinic setting	
Participants	Number randomised: 128 Characteristics: 30.5% women; mean age 23.5 years; all participants smoked ≥ 10 cigarettes per day; mean FTND score 3.9 Specialist population: aged 19–50 years; Japanese ethnicity Inclusion criteria Currently smoke 10–30 cigarettes per day Aged 20–50 years Japanese ethnicity Only smoked Marlboro non‐menthol cigarettes for 2 weeks prior to study Exclusion criteria Unacceptable medical conditions Use of nicotine or tobacco product other than cigarettes within 3 months prior to screening Alcohol or drug problems BMI ≤ 17.6 and ≥ 26.4 kg/m^2^ Use of any medication other than hormonal contraceptives Pregnant or lactating People of childbearing potential who did not agree to use contraception	
Interventions	Randomised (7:7:7:7:4 ratio) to use a EHCSS‐K6 electronic HTP, EHCSS‐K3 electronic HTP, continue smoking Marlboro cigarettes, switch to Lark1 cigarettes, or become abstinent **K6 heated tobacco arm ** Device heating method: electronic Device name: EHCSS‐K6, precursor to THS marketed under brand name IQOS Device manufacturer: PMI Other instructions and details: participants were given tobacco sticks Behavioural support: none mentioned Instructions for smoking cessation/switching: switch entirely to heated tobacco use for study period **K3 heated tobacco arm ** Device heating method: electronic Device name: EHCSS‐K3, precursor to THS marketed under brand name IQOS Device manufacturer: PMI Other instructions and details: participants were given tobacco sticks Behavioural support: none mentioned Instructions for smoking cessation/switching: switch entirely to heated tobacco use for study period **Marlboro cigarette smoking arm** Other instructions and details: participants were given Marlboro cigarettes Behavioural support: none mentioned Instructions for smoking cessation/switching: continue smoking Marlboro cigarettes for study period **Lark1 cigarette smoking arm** Other instructions and details: participants were given Lark1 low machine yield cigarettes Behavioural support: none mentioned Instructions for smoking cessation/switching: switch to smoking Lark1 cigarettes for study period **Abstinence arm** Behavioural support: none mentioned Instructions for smoking cessation/switching: do not smoke cigarettes during study period	
Outcomes	Follow‐up time points: 1 week (8 days) Abstinence outcomes: N/A Safety outcomes: biomarkers of exposure to toxins and carcinogens; biomarkers of harm; adverse events; serious adverse events Prevalence/sales outcomes: N/A	
Notes	**Funding source** Tobacco industry funded: quote: "The work reported in all eight parts of this supplement was funded by PMI R&D". **Author conflicts of interest** Quote: "All authors are or were Philip Morris International (PMI) R&D employees or worked for PMI R&D under contractual agreements".	
***Risk of bias***		
**Bias**	**Authors' judgement**	**Support for judgement**
Random sequence generation (selection bias)	Unclear risk	No details on random sequence generation.
Allocation concealment (selection bias)	Unclear risk	No information.
Blinding of outcome assessment (detection bias) All outcomes	Low risk	All outcomes were biological.
Incomplete outcome data (attrition bias) All outcomes	Unclear risk	No information on attrition.
Selective reporting (reporting bias)	Unclear risk	No trial registration or analysis plan.

**Table:** Tricker 2012c

***Study characteristics***		
Methods	Design: RCT Study dates: not reported Recruitment: no information Location: Belfast, UK Setting: confinement in‐clinic setting	
Participants	Number randomised: 160 Characteristics: 50.0% women; mean age 28.7 years; all participants smoked ≥ 10 cigarettes per day; mean FTND score 5.2 Specialist population: aged 19–50 years Inclusion criteria Currently smoke 10–30 cigarettes per day Aged 20–50 years Only smoked Marlboro non‐menthol cigarettes for 2 weeks prior to study Exclusion criteria Unacceptable medical conditions Use of nicotine or tobacco product other than cigarettes within 3 months prior to screening Alcohol or drug problems Use of any medication other than hormonal contraceptives Pregnant or lactating People of childbearing potential who did not agree to use contraception	
Interventions	Randomised (7:7:7:7:4 ratio) to use a EHCSS‐K6 electronic HTP, EHCSS‐K3 electronic HTP, continue smoking Marlboro cigarettes, switch to Lark1 cigarettes, or become abstinent **K6 heated tobacco arm ** Device heating method: electronic Device name: EHCSS‐K6, precursor to THS marketed under brand name IQOS Device manufacturer: PMI Other instructions and details: participants were given tobacco sticks Behavioural support: none mentioned Instructions for smoking cessation/switching: switch entirely to heated tobacco use for study period **K3 heated tobacco arm ** Device heating method: electronic Device name: EHCSS‐K3, precursor to THS marketed under brand name IQOS Device manufacturer: PMI Other instructions and details: participants were given tobacco sticks Behavioural support: none mentioned Instructions for smoking cessation/switching: switch entirely to heated tobacco use for study period **Marlboro cigarette smoking arm** Other instructions and details: participants were given Marlboro cigarettes Behavioural support: none mentioned Instructions for smoking cessation/switching: continue smoking Marlboro cigarettes for study period **Lark1 cigarette smoking arm** Other instructions and details: participants were given Lark1 low machine yield cigarettes Behavioural support: none mentioned Instructions for smoking cessation/switching: switch to smoking Lark1 cigarettes for study period **Abstinence arm** Behavioural support: none mentioned Instructions for smoking cessation/switching: do not smoke cigarettes during study period	
Outcomes	Follow‐up time points: 1 week (8 days) Abstinence outcomes: N/A Safety outcomes: biomarkers of exposure to toxins and carcinogens; biomarkers of harm; adverse events; serious adverse events Prevalence/sales outcomes: N/A	
Notes	**Funding source** Tobacco industry funded: quote: "The work reported in all eight parts of this supplement was funded by PMI R&D". **Author conflicts of interest** Quote: "All authors are or were Philip Morris International (PMI) R&D employees or worked for PMI R&D under contractual agreements".	
***Risk of bias***		
**Bias**	**Authors' judgement**	**Support for judgement**
Random sequence generation (selection bias)	Unclear risk	No details on random sequence generation.
Allocation concealment (selection bias)	Unclear risk	No information.
Blinding of outcome assessment (detection bias) All outcomes	Low risk	All outcomes were biological.
Incomplete outcome data (attrition bias) All outcomes	Unclear risk	No information on attrition.
Selective reporting (reporting bias)	Unclear risk	No trial registration or analysis plan.

BMI: body mass index; FEV_1_: forced expiratory volume in one second; FTND: Fagerstrom Test for Nicotine Dependence; FVC: forced vital capacity; HTP: heated tobacco product; N/A: not available/applicable; PMI: Philip Morris International; RCT: randomised controlled trial.

### Characteristics of excluded studies [ordered by study ID]

**Table:**

Study	Reason for exclusion
Adriaens 2018	Insufficient follow‐up length.
Dei Giudici 2019	Wrong study design as it only assessed acute effects of heated tobacco.
Franzen 2020	Wrong study design as it only assessed acute effects of heated tobacco.
Gale 2017	Wrong study design as participants used heated tobacco product for < 7 days.
Haziza 2016b	Wrong study design as participants used heated tobacco product for < 7 days.
Ioakeimidis 2021	Wrong study design as it only assessed acute effects of heated tobacco.
Lee 2020	Wrong study design and outcome as formal interrupted or multiple time‐series not used to assess change in trends in smoking prevalence or cigarette sales.
Martin 2016	Wrong study design as participants used heated tobacco product for < 7 days.
Pataka 2019	Wrong study design, as it only measures acute effects of heated tobacco use.
Tran 2020	Wrong study design as participants used heated tobacco product for < 7 days.
Yuki 2018	Wrong study design as participants used heated tobacco product for < 7 days.

### Characteristics of ongoing studies [ordered by study ID]

**Table:** Caponnetto 2020

Study name	Non‐inferiority trial comparing cigarette consumption, adoption rates, acceptability, tolerability, and tobacco harm reduction potential in smokers switching to heated tobacco products or electronic cigarettes: study protocol for a randomized controlled trial
Methods	Design: RCT Study dates: May 2019 to May 2020 Recruitment: advertising on social networks, in local media, and through distribution of flyers at university campus Location: Catania, Italy Setting: ambulatory setting
Participants	Number randomised: 220 expected Characteristics: N/A Specialist population: aged ≥ 19 years Inclusion criteria Healthy Current smoker (≥ 10 cigarettes per day) Smoker for ≥ 1 year Aged ≥ 19 years No intention to quit smoking in next 30 days Exclusion criteria Used nicotine product other than cigarettes or smoking cessation medication in past 3 months Pregnant or breastfeeding
Interventions	Randomised (1:1 ratio) to use an electronic HTP or an electronic cigarette **Heated tobacco arm** Device heating method: electronic Device name: IQOS 2.4 Device manufacturer: PMI Other instructions and details: receive IQOS 2.4 and tobacco sticks of their choice, with 3 flavour options. No products given past 12 weeks' follow‐up point Behavioural support: trained and counselled on use of HTP. Reports offered to participants at 12 weeks' follow‐up to minimise risk of relapse to smoking Instructions for smoking cessation/switching: switch from smoking cigarettes to using HTP **E‐cigarette arm** Device name: Just Fog Starter Kit Device Manufacturer: JFT Co Other instructions and details: receive Just Fog Starter Kit and e‐liquid of their choice, with 3 flavour options. No products given past 12 weeks' follow‐up point Behavioural support: trained and counselled on use of e‐cigarette. Reports offered to participants at 12 weeks' follow‐up to minimise risk of relapse to smoking Instructions for smoking cessation/switching: switch from smoking cigarettes to using e‐cigarette
Outcomes	Follow‐up time points: 1, 2, 4, 8, 12, and 24 weeks Abstinence outcomes: carbon monoxide‐verified abstinence from tobacco smoking Safety outcomes: biomarkers of toxicant and carcinogen exposure; biomarkers of harm; adverse events; serious adverse events Prevalence/sales outcomes: N/A
Starting date	May 2019
Contact information	Name: Pasquale Caponnetto, PhD Email: p.caponnetto@unict.it
Notes	**Funding source** Tobacco industry funded: quote: "This research is supported by an Investigator‐Initiated Study award by Philip Morris Products SA (PMI.IIS.2016.006). The study protocol was written by EM who was also the principal investigator of the study. Philip Morris Products SA had no role in the design of the study protocol and will not have any role during its execution, analysis, data interpretation or writing of the manuscript". **Author conflicts of interest** Quote: "EM, DS and RP are full‐time employee of the University of Catania, Italy. PC, MC and RE are fixed‐term researcher at University of Catania, Italy. MM is fixed‐term researcher at Centro per la Prevenzione e Cura del Tabagismo, University of Catania. BB is full‐time employee of ARNAS Garibaldi, Catania, Italy. AP is full‐time employee of Casa di Cura Musumeci‐Gecas, Gravina di Catania, Italy. UP is full‐time employee of Ospedale “San Vincenzo" – Taormina, Italy. In relation to his work in the area of tobacco control, RP has received lecture fees and research funding from Pfizer and GlaxoSmithKline, manufacturers of stop smoking medications. He has also received support from The Consumer Advocates for Smoke‐free Alternatives (CASAA) for publication and open access costs of one paper. He has also served as a consultant for Pfizer, Global Health Alliance for treatment of tobacco dependence, ECITA (Electronic Cigarette Industry Trade Association, in the UK), Arbi Group Srl., and Health Diplomats (consulting company that delivers solutions to global health problems with special emphasis on harm minimization). Lectures fees from a number of European electronic cigarette industry and trade associations (including FIVAPE in France and FIESEL in Italy) were directly donated to vapers advocacy no‐profit organizations. He is also currently involved in the following pro bono activities: scientific advisor for LIAF, Lega Italiana Anti Fumo (Italian acronym for Italian Anti Smoking League) and for The Consumer Advocates for Smoke‐free Alternatives (CASAA); Chair of the European Technical Committee for standardization on "Requirements and test methods for emissions of electronic cigarettes" (CEN/TC 437; WG4). The other authors have no conflict of interests to declare".

**Table:** NCT03837704

Study name	Comparison of abdominal aortic aneurysm growth in adult smoking patients who either switch to IQOS, continue smoking, or quit smoking
Methods	Design: RCT Study dates: October 2018 to April 2025 Recruitment: no information Location: Atsugi, Japan Setting: unclear if confinement in‐clinic or ambulatory setting
Participants	Number randomised: 114 expected Characteristics: N/A Specialist population: aged ≥ 50 years; diagnosed with abdominal aortic aneurysm Inclusion criteria Currently smoke ≥ 10 cigarettes per day Smoked cigarettes for past ≥ 12 months Aged ≥ 50 years No intention to quit smoking within next 6 months Diagnosed with abdominal aortic aneurysm Exclusion criteria Medical conditions that were a safety concern or would interfere with study Use drugs likely to interfere with study Current or past alcohol or drug problems Pregnant or breastfeeding People of childbearing potential who do not agree to use contraception Current or past employee of tobacco industry (or their close relatives)
Interventions	Randomised to use an electronic HTP or continue smoking cigarettes **Heated tobacco arm** Device heating method: electronic Device name: IQOS Device manufacturer: PMI Other instructions and details: ad libitum use with no flavour restrictions Behavioural support: no information Instructions for smoking cessation/switching: switch entirely to IQOS for study period **Cigarette smoking arm** Behavioural support: no information Instructions for smoking cessation/switching: not advised to stop smoking
Outcomes	Follow‐up time points: 5 years Abstinence outcomes: N/A Safety outcomes: biomarkers of toxicant and carcinogen exposure; biomarkers of harm; adverse events; serious adverse events Prevalence/sales outcomes: N/A
Starting date	October 2018
Contact information	Name: Christelle Haziza, PhD Email: christelle.haziza@pmi.com
Notes	**Funding source** Assumed to be tobacco industry funded, as PMI is the study sponsor. **Author conflicts of interest** No conflicts of interest statement available.

**Table:** NCT03887117

Study name	Effect of switching from cigarette smoking to IQOS on exercise capacity
Methods	Design: RCT Study dates: February 2019 to March 2020 Recruitment: no information Location: Mannheim, Germany Setting: ambulatory setting
Participants	Number randomised: 93 expected Characteristics: N/A Specialist population: none Inclusion criteria Healthy Currently smoke ≥ 10 cigarettes per day Smoked menthol cigarettes for past ≥ 12 months No plan to quit smoking within next 6 months Exclusion criteria Medical conditions that were a safety concern or would interfere with study BMI < 18.5 or > 32.0 kg/m^2^ Use of nicotine‐containing products other than menthol cigarettes in 4 weeks prior to screening Performs < 45 minutes of vigorous exercise per week Use drugs likely to interfere with study Current or past alcohol problems Positive urine drug test Pregnant or breastfeeding People of childbearing potential who do not agree to use contraception Current or past employee of tobacco industry (or their close relatives)
Interventions	Randomised to use an electronic HTP (with or without an exercise program), continue smoking cigarettes, or become abstinent **Heated tobacco with exercise training arm ** Device heating method: electronic Device name: IQOS Device manufacturer: PMI Other instructions and details: asked to buy their own tobacco sticks Behavioural support: exercise training programme Instructions for smoking cessation/switching: switch entirely to IQOS for study period **Heated tobacco without exercise training arm ** Device heating method: electronic Device name: IQOS Device manufacturer: PMI Other instructions and details: asked to buy their own tobacco sticks Behavioural support: no information Instructions for smoking cessation/switching: switch entirely to IQOS for study period **Cigarette smoking arm** Behavioural support: no information Instructions for smoking cessation/switching: not advised to stop smoking **Abstinence arm** Behavioural support: no information Instructions for smoking cessation/switching: do not smoke cigarettes during study period
Outcomes	Follow‐up time points: 1, 6, 10, and 15 weeks Abstinence outcomes: N/A Safety outcomes: biomarkers of toxicant and carcinogen exposure; biomarkers of harm Prevalence/sales outcomes: N/A
Starting date	February 2019
Contact information	Name: Christelle Haziza, PhD Email: christelle.haziza@pmi.com
Notes	**Funding source** Assumed to be tobacco industry funded, as PMI is the study sponsor. **Author conflicts of interest** No conflicts of interest statement available.

HTP: heated tobacco product; N/A: not available/applicable; PMI: Philip Morris International; RCT: randomised controlled trial.

## Differences between protocol and review

We originally stated that we would include the term "pulse" in literature searches, but we intended to refer to the heated tobacco brand "pulze"  (Tattan‐Birch 2020). We corrected this typographical error.

Our protocol did not specify how we would pool studies reporting mixtures of arithmetic and geometric means (Tattan‐Birch 2020). We updated the methods to note that, following guidance from Higgins 2008, we would convert all results onto the natural log scale before pooling.

Our protocol specified that, when assessing safety, we would only include studies with at least seven days' follow‐up (Tattan‐Birch 2020). Here, we also only included studies where participants in the heated tobacco products (HTP) arm were instructed to stop smoking combustible cigarettes for at least seven days, because shorter lengths of smoking abstinence were deemed inadequate for judging comparative safety.

Alongside our primary outcomes, we included two biomarkers of exposure, NNAL and carboxyhaemoglobin, in the summary of findings tables. Both biomarkers are well studied and validated indicators of exposure to tobacco smoke, and NNAL is highly correlated with other toxicants and carcinogens produced by combustion (Chang 2017; Joseph 2005). In the absence of data on smoking cessation, they provide an indication of whether HTPs expose users to fewer harmful chemicals than cigarettes.

Our protocol noted that we would assess performance bias in included randomised controlled trials (Tattan‐Birch 2020). When applied to behavioural interventions, such as smoking cessation treatment, risk of performance bias is often assessed by judging whether the level of behavioural support provided was similar in treatment and control arms. The literature search did not identify any studies reporting on smoking cessation, and assessment of performance bias was deemed less relevant for safety outcomes. Therefore, we did not evaluate risk of performance bias. 

In our protocol, we said we would conduct sensitivity analyses where we only included studies reporting per‐protocol results for biomarker outcomes (i.e. only including participants who exclusively used the product they were assigned to) (Tattan‐Birch 2020). We instead reported subgroup analyses for per‐protocol and intention‐to‐treat results. We also added sensitivity analyses where we removed studies that gave participants carbon‐tip HTPs, as these products are not widely available in most countries. 
